# Identification of *oriT* and a recombination hot spot in the IncA/C plasmid backbone

**DOI:** 10.1038/s41598-017-11097-0

**Published:** 2017-09-06

**Authors:** Anna Hegyi, Mónika Szabó, Ferenc Olasz, János Kiss

**Affiliations:** Agricultural Biotechnology Institute, National Agricultural Research and Innovation Centre, 4. Szent-Györgyi Albert str., Gödöllő, Hungary

## Abstract

Dissemination of multiresistance has been accelerating among pathogenic bacteria in recent decades. The broad host-range conjugative plasmids of the IncA/C family are effective vehicles of resistance determinants in Gram-negative bacteria. Although more than 150 family members have been sequenced to date, their conjugation system and other functions encoded by the conserved plasmid backbone have been poorly characterized. The key cis-acting locus, the origin of transfer (*oriT*), has not yet been unambiguously identified. We present evidence that IncA/C plasmids have a single *oriT* locus immediately upstream of the *mobI* gene encoding an indispensable transfer factor. The fully active *oriT* spans ca. 150-bp AT-rich region overlapping the promoters of *mobI* and contains multiple inverted and direct repeats. Within this region, the core domain of *oriT* with reduced but detectable transfer activity was confined to a 70-bp segment containing two inverted repeats and one copy of a 14-bp direct repeat. In addition to *oriT*, a second locus consisting of a 14-bp imperfect inverted repeat was also identified, which mimicked the function of *oriT* but which was found to be a recombination site. Recombination between two identical copies of these sites is RecA-independent, requires a plasmid-encoded recombinase and resembles the functioning of dimer-resolution systems.

## Introduction

The discovery and use of antibiotics have helped to save millions of lives in recent decades^1^. The overuse of antibiotics, however, has contributed to the emergence of new multi-resistant pathogens worldwide, leading to considerable public health problems. Dissemination of antibiotic resistance traits among different bacterial species mainly occurs via conjugation. Thus, understanding this DNA transfer mechanism evokes general interest. The process of conjugation starts with the assembly of a multi-protein complex called the relaxosome around the origin of transfer (*oriT*), the only DNA sequence required *in cis* for DNA transfer (for reviews see refs 2–4). *OriT* is often located near genes encoding relaxosome components (e.g., in plasmids RP4, R388, R6K, pCW3, pIP501). In many cases, it contains inverted repeat motifs and AT-rich regions as observed in F^5^, R6K^6^, IncPα^7, 8^, pAD1^9^, pAM373^9^ or pCW3^10^ plasmids. Conserved sequence motifs have been found in *oriT*s close to the *nic* site of several conjugative plasmids such as IncPα plasmids, Ti and Ri plasmids, R64 and pTF-FC2^11^. Relaxase (TraI), the key enzyme of the relaxosome, cuts either strand of *oriT* DNA at the *nic* site and covalently binds to the 5′ end of the cleaved strand. The relaxase-DNA complex is subsequently transferred across the membrane-associated DNA transport machinery, the type IV secretion system (T4SS), to the recipient. In addition to TraI, conjugation may require several auxiliary proteins. For example, TraJ of the IncPα plasmid RP4 is indispensable for the initiation of transfer because it binds *oriT* at a specific site^12^ and directs the relaxase to the *nic* site. The complex of *oriT* DNA, TraJ and TraI is stabilized by TraH through specific protein-protein interactions^13^. TrwA of the IncW plasmid R388 also binds specifically to *oriT* and stimulates the ATPase activity of the coupling protein TrwB^14^, which is required to link the relaxosome complex to T4SS^15, 16^.

Large single-copy conjugative plasmids of the IncA/C family are often associated with resistance to several antimicrobial agents. Owing to their broad host range, an effective conjugative transfer system and the ability to mobilize multidrug-resistant genomic islands, IncA/C plasmids efficiently distribute multidrug resistance phenotypes among Gram-negative bacteria. Comparative genomic studies have revealed that IncA/C plasmids have a highly conserved backbone that is interrupted by variable antibiotic resistance islands (ARI) at specific positions^17^. Conjugation, maintenance and replication functions are encoded in the backbone, while mobile genetic elements, such as transposons and integrons, which often carry resistance genes, are localized to ARIs. Similarly to the IncH, J, T and P7 groups of plasmids, as well as the SXT family of integrative conjugative elements (ICEs), the conjugative transfer system of IncA/C plasmids has been classified into the MOB_H_ family^18^. The transfer genes (relaxosomal and T4SS genes), mostly identified by their homology to characterized *tra* genes of other systems, are encoded in two clusters. The first region contains genes encoding the putative relaxase (*traI*), coupling protein (*traD*), mating pair stabilization protein (*traN*) and proteins implicated in T4SS assembly (*traLEKBVACWU*). The second cluster encodes three additional putative proteins involved in T4SS assembly (*traFHG*)^17^. Moreover, this region contains two ORFs encoding the FlhDC-family transcriptional activator AcaCD, which activates the expression of transfer genes^19^ and, therefore, is essential for the conjugation of the IncA*/*C plasmids. Near the *acaCD*, another key transfer gene, *mobI*, has also been identified, although its exact role has not yet been established^20^. IncA/C-encoded MobI shares 28% identity with MobI protein of SXT/R391 ICEs^20^, which is indispensable for the transfer of SXT/R391 and is implicated in the recognition of *oriT*_SXT_^21^.

Biochemical characterization of the relaxase, the MobI or the initiation complex of IncA/C plasmids has not been conducted to date, and the exact position of *oriT* sequence has not been determined, either. Originally, IncA/C *oriT* was predicted to be located near *traD*, however, this annotation was not confirmed experimentally^22, 23^. Based on the homology of the regions surrounding *oriT* of SXT/R391 with the corresponding region of the IncA/C plasmid pVCR94, *oriT* was proposed to be within the intergenic region of *vcrx001* (*mobI*) and *vcrx152* ^20^. Although this region was confirmed to carry a functional *oriT*, two different deletions within the predicted *oriT* sequence caused only a 10-fold reduction in the transfer frequency, but they did not abolish conjugation. Thus, as a plausible explanation, the presence of a second *oriT* was suggested. Although most conjugative plasmids possess a single *oriT*, dual *oriT* systems have also been reported in the cases of ICE*clc* of *Pseudomonas knackmussi* B13^24^ or plasmids pAD1^9, 25^ and R6K^6^, which might support the assumption.

Here, we show that IncA/C plasmids have a single *oriT* locus that is situated closer to the *mobI* gene than previously predicted^20^. The ca. 150-bp AT-rich *oriT* sequence containing multiple short inverted and 14-bp partially overlapping direct repeat motifs covers the promoter region of *mobI*. The core domain of *oriT* is confined to a 70-bp segment containing only two inverted repeats (IR) and one copy of the 14-bp direct repeat (DR). We have also identified another locus that appeared to be a second *oriT* in our experimental setup, but instead was found to be a recombination site consisting of a 14-bp imperfect inverted repeat. Recombination between two identical copies of this putative recombination site, resembling the *res* sites of several site-specific dimer-resolution systems, is RecA-independent and requires a plasmid-encoded recombinase. Deletion mutants generated in *oriT*, *mobI* and the putative relaxase gene caused transfer deficiency, which validated their indispensability for IncA/C conjugation. However, the recombination site did not appear to be involved in transfer. Sequence comparisons indicate that the recombination site is well conserved, suggesting that its intactness is important for IncA/C plasmids in the evolutionary timescale.

## Results

### Exploration of *oriT* on IncA/C plasmids

*OriT* of several conjugative plasmids was found adjacent to genes encoding relaxosome components and *oriT* of IncA/C plasmids was also predicted to be near *traI*. To prove this assumption, *traI* of R16a and IP40a was knocked out using the one-step gene inactivation method^26^. These IncA/C plasmids were chosen because they have few resistance markers^27^, which makes it easier to apply this KO technique. Due to the lack of sequence information at that time, KO experiments were designed according to the sequence of R55, which has been previously published^28^ and reported to be a close relative of R16a and IP40a^27^. The *traI* KO mutants, as expected, could not conjugate, confirming that this gene is indispensable for IncA/C transfer. The Cm^R^ marker replacing *traI* in the KO mutants enabled us to clone the region surrounding *traI*. Accordingly, the *Hpa*I fragment of R16a Δ*traI*::*cat* mutant containing >4.2 kb flanking regions of both sides of *traI* was inserted into a non-mobilizable pACYC177-derivative^29^ vector, pJKI708. Transfer of the resulting plasmid, pJKI854, was tested using *E*. *coli* donor strain TG1Nal/R16a. While conjugation of R16a into Tc^R^ recipient *E*. *coli* strain TG2 occurred with a frequency of 3.5 ± 1.2 × 10^−4^, transfer of pJKI854 was undetectable (<1.1 ± 0.9 × 10^−7^), indicating that functional *oriT* was missing not only in the region between *traD* and *traJ*, as shown previously^20^, but also in the ca. 11.6-kb region of the 12 ORFs including a *topoisomerase III* gene, *traI*, *traD*, the conjugative transfer protein genes *234* and *s043(traJ)*, as well as several other hypothetical genes.

**Figure 1:**
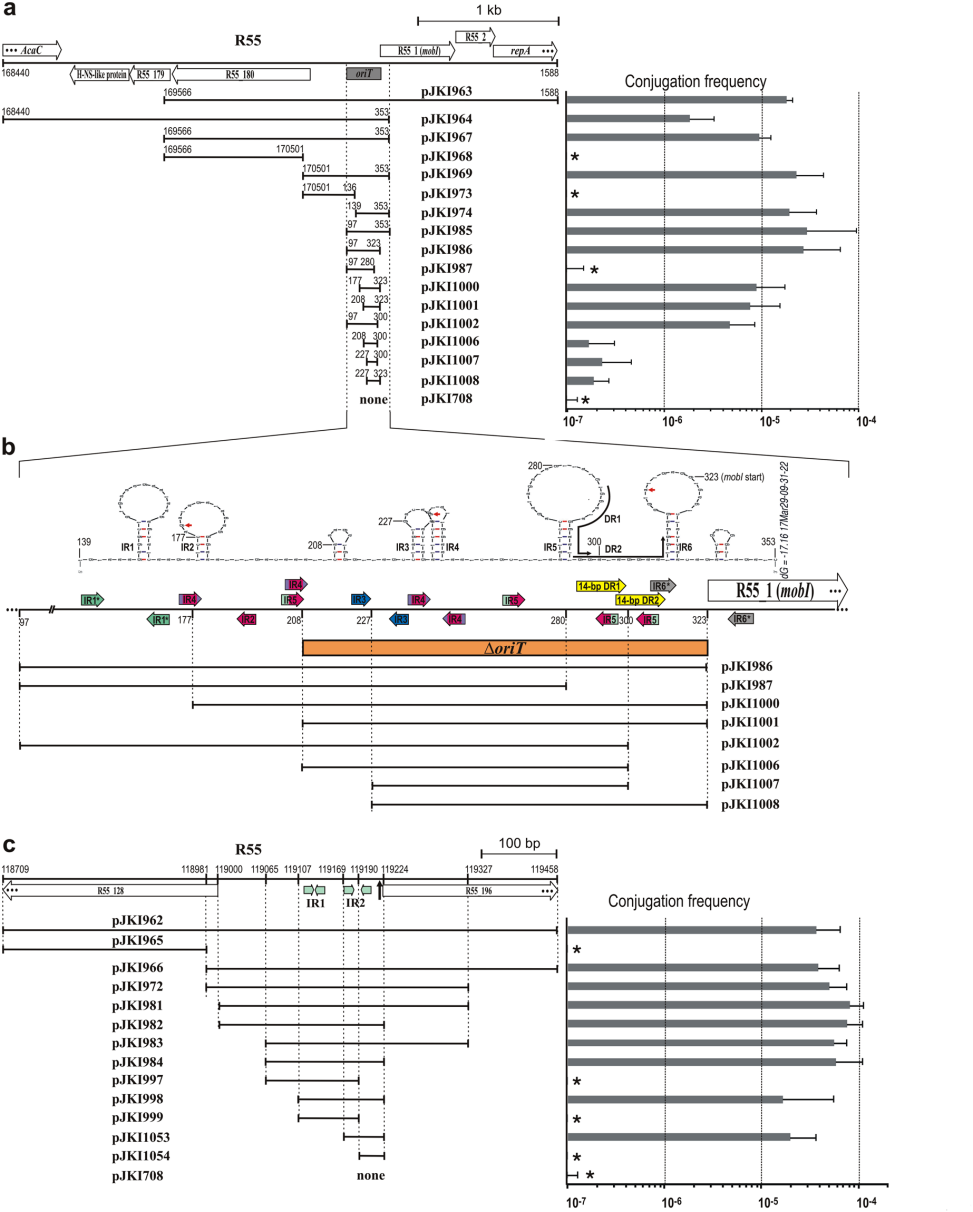
Identification of mobilizable fragments of R55. Subclones of two Mob regions were constructed in the p15A-based vector pJKI708, and their conjugation frequency was measured in the presence of R55 using the *recA E*. *coli* strain TG2. pJKI708 (without insert) was used as a negative control. The schematic maps representing the regions of R55 covered by the subclones listed below are drawn to scale, and the coordinates are shown according to the published R55 sequence. Open arrows indicate the annotated ORFs. Stretches with coordinates represent the fragments carried by the p15A-based plasmids. Asterisks indicate that the transfer frequency was below the detection limit (<10^−8^). (**a**) Conjugation frequency of different subclones of Mob 1 region. The grey box shows the *oriT* region. (**b**) Detailed map of the *oriT* region. Color-coded arrows represent inversely (IR) and directly (DR) repeated sequence motifs of at least 4 bp in length (IR1: 6 bp with 1 mismatch, IR2: 4 bp, IR3: 5 bp, IR4: 6 bp, IR5: 6 bp and IR6: 7 bp with 1 mismatch). Asterisks in IR1 and IR6 refer to the imperfect repeat. The red region in IR4 and IR5 represents the 4-bp IR2 motif as a part of these repeats. The region deleted from R16a and IP40a (resulting in the Δ*oriT* mutants) and the p15A-based subclones of R55 *oriT* region are shown below the graph. The potential secondary structure of the region is also shown. Coordinates corresponding to the endpoints of cloned fragments and the repeated motifs are indicated. The red arrows point to the base positions where IncA/C family plasmids most frequently carry divergent bases (see also Supplementary Fig. 6). (**c**) Transfer frequency of subclones of the Mob 2 region. The 11-bp IR1 and 14-bp IR2 inverse repeat motifs are shown as light green arrows. Vertical arrows indicate the positions of ARI_R16a_ and Tn6333 insertions in the related R16a and IP40a plasmids.

To identify *oriT*, nine different libraries were constructed from R55 in vector pJKI708 and introduced into *E*. *coli* strain TG2/R55. The *recA* strain was applied to reduce homologous recombination between R55 and the cloned R55 fragments. Using these transformant cell populations as donors in crosses with the TG1Nal recipient strain, transconjugants were obtained from three libraries. In the case of libraries L1, L2 and L5, the frequency of transconjugants was 4.2 × 10^−9^, 1.9 × 10^−9^ and 2.0 × 10^−6^, respectively. Extensive restriction analysis of the transferred plasmid species revealed two different mobilizable regions (Mob 1 and 2) in R55. Among the transferred clones obtained from L2, the shortest insert (2.72 kb) proved to be part of all the longer clones from L1 and L2 and spanned the ORFs from *R55_176* to the upstream intergenic region of *R55_180* and the 5′ portion of ORF *R55_1* (pJKI964, Fig. 1a). By contrast, two different mob regions were found in the L5 library. One of these clones, named pJKI963, contained a 2.83-kb fragment that partially overlapped the insert of pJKI964 and contained ORFs from *R55_180* up to the 5′ part of *R55_3* (*repA*). The overlapping part of these clones was named the Mob 1 region (pJKI967). These results were consistent with that the *oriT* of another IncA/C plasmid, pVCR94, has previously been localized in this region^20^. The other mobilized clone from L5, pJKI962, contained a 0.75-kb *Sac*I fragment named Mob 2, which contained the intergenic region flanked by the truncated ORFs *R55_128* and *R55_196* (Fig. 1c).

The identification of minimal functional *oriT*s in the Mob 1 and the Mob 2 regions was based on the mobilization assays for the three original clones and their gradually shortened derivatives created by repeated subcloning (Fig. 1). All but one plasmid carrying the Mob 1 fragment comprising the 139–323 bp region of R55 showed a maximal transfer rate. The only exception, pJKI964, contained the H-NS-like protein gene *acr2*, which was identified as a repressor of *acaCD*, encoding the master activator of *tra* genes^19^. The lower conjugation frequency of pJKI964 (and also that of the R55 helper plasmid, data not shown) can be explained by the elevated Acr2 level produced by the 15–20 copies of the p15A-based pJKI964 in the donor cells. All these data suggested that the fully functional minimal *oriT* sequence in the Mob 1 region was located between the 139–323 bp positions within the intergenic region of the divergent ORFs *R55_180* and *R55_1* (*mobI*) (corresponding to the overlapping region of pJKI974 and pJKI986). Testing the Mob 2 region by a similar method indicated that the putative second *oriT* is situated in a short fragment (119169–119224 bp) surrounded by the divergent ORFs *R55_128* and *R55_196* (pJKI1053).

### Identification and characterization of *oriT* in the Mob 1 region

Sequence analysis of the Mob 1 region revealed a complex array of short inverted and direct repeats (IRs and DRs, respectively). For a more exact localization of the functional *oriT* sequence, the Mob 1 region of R55 was further shortened by deleting the repetitive motifs (Fig. 1a,b). The transfer frequencies of these subclones showed that the absence of the 97–139 bp segment (pJKI986/974) had no effect, while the lack of the sequence containing IR1 and IR2 (pJKI1000/1001) slightly reduced the mean transfer rate values. By contrast, the deletion of the overlapping 14-bp DRs located within the 280–323 bp segment (pJKI987) completely abolished the transfer, although the deletion of a single copy of this DR had only a weak impact (pJKI1002). The deletion of the 208–227 bp segment, including IR1, IR2 and one copy of IR3 (pJKI1008), or the simultaneous removal of the 97–208 bp and 300–323 bp regions (pJKI1006), significantly reduced the transfer frequency. The shortest fragment that had only the IR4, IR5 and one copy of the 14-bp DR (pJKI1007) showed a similarly low, but still detectable, transfer rate. These data again confirmed that the fully functional *oriT* was located between the 139–323 bp segment of R55, while a minimal functional *oriT* was found within the 227–300 bp region; however, this sequence already lacks several important sequence motifs.

To further analyze the function of this region, we created deletion mutants of our IncA/C plasmids. R55 was not the best candidate for gene KO due to its multiple resistance genes, therefore R16a and IP40a were used instead. To verify that the *oriT* region was similar or identical in all three plasmids, the *mobI* genes and the segments corresponding to 97–399 bp of the Mob 1 region in R55 were amplified and sequenced from R16a and IP40a. The *mobI* genes of the three plasmids and the *oriT* region of R55 and IP40a were identical, while the only sequence divergence in the *oriT* region of R16a was the presence of an additional copy of the 14-bp DR. After cloning this divergent *oriT* fragment, its conjugation was compared to that of the analogous R55 fragment from donor strains containing either R55 or R16a helper plasmids (Supplementary Table 1). The results indicated that both fragments were similarly mobilized by both helpers, which confirmed the equivalence of these sequence variants and that the planned KO mutations in R16a and IP40a would provide general information regarding the function of this region. Consequently, the *mobI* gene and its 126-bp upstream region were independently deleted in R16a and IP40a by the one-step gene KO method. Surprisingly, both mutations resulted in the cessation of conjugation as the transfer frequencies of the Δ*oriT* and Δ*mobI* mutants of both plasmids were below the detection limit (<6.9 ± 5.2 × 10^−8^), while those of wt R16a and IP40a were 7.6 ± 4.8 × 10^−3^ and 1.0 ± 0.7 × 10^−4^, respectively. The complete termination of transfer by the deletion of either *oriT* or *mobI* indicated that the intact Mob 2 region could not complement the deleterious effects of these KO mutations in the Mob 1 region.

**Figure 2:**
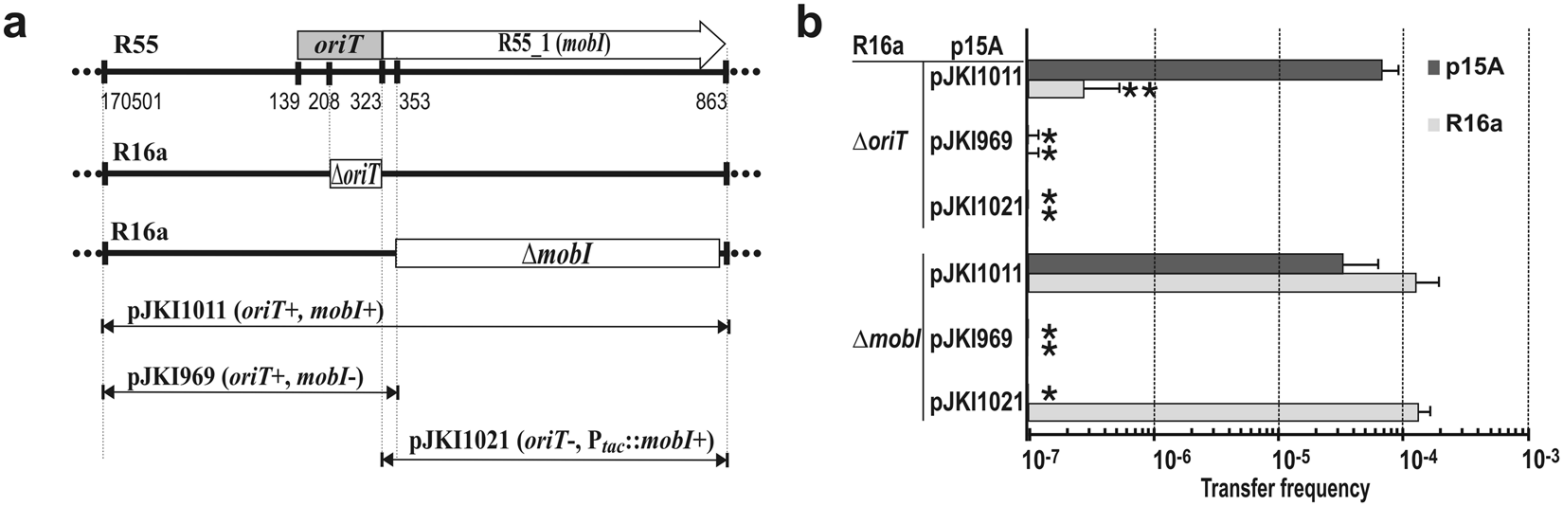
Complementation of deletion mutants in the Mob 1 region. (**a**) Schematic map of deletion mutants generated in the Mob 1 region of R16a (thick line) and the regions cloned from R55 into a p15A-based non-mobile vector (thin arrows) that were applied in complementation tests. Open boxes represent the deleted regions in R16a (the same deletions were also created in IP40a), and other symbols are as described in Fig. 1.(b) Conjugation assays were performed to demonstrate the trans-mobilization effect of the different fragments of the R55 Mob 1 region on the transfer-defective R16a deletion mutants. The bars show the mean transfer frequencies of the Δ*oriT* and Δ*mobI* R16a mutants and the complementing p15A-based plasmids when present together in the donor TG1Nal *E*. *coli* cells. ‘*’Indicates that the transfer frequency was below the detection limit (<10^−8^). ‘**’Indicates a very low transfer frequency of Δ*oriT* R16a when the complementing plasmid carried *oriT*+*mobI*. In this case, co-transfer of the two plasmids was >67%.

As *oriT* in the Mob 1 region is located immediately upstream of the *mobI* gene, which is essential for conjugation^20^, we supposed that the *oriT* deletion knocked down *mobI* expression by removing its promoter. To test this hypothesis, complementation of the KO mutant IncA/C plasmids was carried out using three p15A-based plasmids containing the Mob 1 region with or without an intact *mobI* gene (pJKI1011 and pJKI969, respectively) and an expression vector, pJKI1021, carrying *mobI* under the control of the P_*tac*_ promoter without the *oriT* region (Fig. 2a). Plasmids pJKI1011 (*oriT* ^+^, *mobI* ^+^) and pJKI1021 (*oriT* ^−^, *mobI* ^+^) expressing *mobI in trans* restored the transfer of the Δ*mobI* R16a mutant, and pJKI1011 could also be transferred, as expected. However, none of the plasmids could complement the Δ*oriT* R16a mutant, indicating that the deletion inactivated the *oriT* sequence itself and that the transfer deficiency in this case was not due to the lack of MobI protein (Fig. 2b). Similar results were obtained for IP40a (data not shown), but all transfer frequencies were ca. two orders of magnitude lower, as also observed for the wt plasmid. The vast majority of the few R16a Δ*oriT* transconjugants, with a frequency near the detection limit (ca. 5.0 × 10^−7^), also contained pJKI1011, which suggested that R16a transfer occurred via R16a::pJKI1011 cointegrates formed possibly via homologous recombination. In these cointegrates both *oriT* and MobI could be provided by pJKI1011. By contrast, transconjugants were not obtained in the presence of pJKI969 (*oriT* ^+^, *mobI* ^−^) despite the similar possibility of plasmid cointegrate formation. The complete lack of transfer of both the *oriT* ^+^ plasmid pJKI969 and the Δ*oriT* R16a helper clearly supported the conclusion that the deletion of *oriT* also prevented *mobI* expression. The results also strengthened the previous observation that the intact Mob 2 region could not complement the Δ*oriT* mutation even in the presence of MobI. All these findings confirmed that IncA/C plasmids have a single *oriT* that is located in the Mob 1 region immediately upstream of the *mobI* gene.

### The promoter region of *mobI* overlaps *oriT*

**Figure 3:**
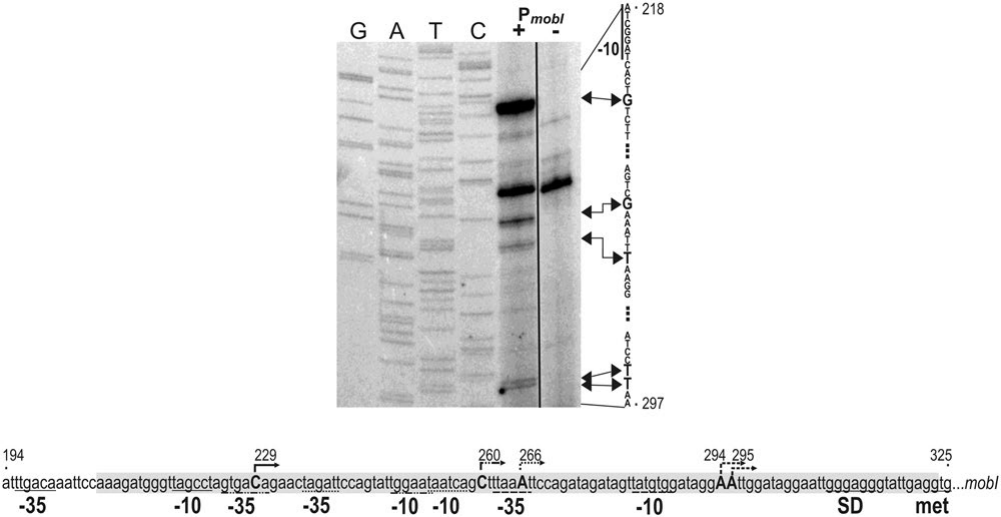
Determination of *mobI* TSS. The primer extension reaction was performed using total RNA purified from *E*. *coli* TG1 cells carrying the P_*mobI*_::*lacZ* fusion plasmid pMSZ952 (+) or pJKI990 as a negative control (−). Primer pUCfor21 annealing near the start codon of *lac*Z was used for extension reactions and sequencing. Lanes G, A, T, C: Sanger sequencing reactions obtained with pMSZ952 template DNA. Arrowheads denote the bases corresponding to the TSSs on the non-transcribed strand. The arrows with R55 coordinates above the sequence of the sense strand show the detected TSSs. The start codon and the deduced Shine-Dalgarno, −10 and −35 boxes in the P_*mobI*_ region are indicated below the sequence. The line styles of the arrows and underlining of the promoter boxes indicate the respective TSS, −10 and −35 box. The deleted region in the Δ*mobI* R16a and IP40a mutants is highlighted in grey. The full-length gel is presented in Supplementary Fig. 1.

The non-coding region between the divergent genes *R55_180* and *mobI* was inserted into a β-galactosidase tester plasmid to measure the promoter activity that drives *mobI* expression. In the resulting plasmid pMSZ952, the upstream region of *mobI* was fused to the promoterless *lacZ* gene. The original GTG start codon of *mobI* was replaced with ATG of *lacZ*. The β-gal assay showed that this region contains a relatively strong promoter as pMSZ952 produced 2462±55 U of β-galactosidase, while the empty vector pJKI990 used as a negative control produced 1.0±0.5 U. For further specification of this promoter, the transcription start site (TSS) of *mobI* was determined in a primer extension experiment (Fig. 3). Based on the strongest signal, the TSS was located 93 bp upstream of the GTG start codon, however, several weaker signals were also obtained compared to the negative control. The promoter identified by the primary TSS has a TTGACA −35 box corresponding with the σ^70^ consensus and a less obvious −10 box. All secondary TSSs were closer to the start codon, and the respective promoter-like sequences appeared to be more divergent from the consensus. Our results, however, indicated that all possible promoters of *mobI* were located in the core region of *oriT*.

### MobI protein is a plasmid-specific transfer factor

MobI has been shown to be indispensable for IncA/C conjugation, although its exact function is not yet clear. As IncA/C plasmids are the exclusive and efficient conjugation helpers of the multiresistant mobilizable integrative elements such as *Salmonella* Genomic Island 1 (SGI1) and its variants, we tested whether MobI was involved in SGI1 transfer. The R16a Δ*mobI* mutant was applied in a SGI1 mobilization assay from *E*. *coli* strain TG1Nal::SGI1-C, which showed that the conjugation-deficient Δ*mobI* plasmid could mobilize SGI1 more efficiently than wt R16a (Table 1). The higher rate of SGI1 transfer observed in the case of the Δ*mobI* helper plasmid may be explained by the lack of competition between SGI1 and the helper for components of the transfer apparatus as the mutant plasmid is unable to conjugate probably due to an error in the initiation. This result clearly indicated that MobI is a plasmid-specific factor that is not required for SGI1 transfer.

**Table 1:** Mobilization of SGI1 by R16a Δ*mobI* mutant from TG1Nal::SGI1-C donor into TG2 recipient strain.

Helper	Conjugation frequency^a^ of	
	Helper plasmid	SGI1-C
R16a wt	1.4 ± 0.68 × 10^−3^	7.8 ± 4.2 × 10^−5^
R16a Δ*mobI*	<3.5 ± 0.82×10^−6b^	3.5 ± 0.72 × 10^−3^

^a^Conjugation frequency is given as transconjugant/donor titer.

^b^Transfer of R16a Δ*mobI* was undetectable.

### The Mob 2 region contains a recombination hot spot rather than a second *oriT*

The first suggestion that the Mob 2 region may not contain a real *oriT* derived from the analysis of KO mutants of the Mob 1 region. If a second, active *oriT* was present in the Mob 2 region, the elimination of *oriT* in Mob 1 should not have abrogated the plasmid transfer. By contrast, the deletion of *oriT* in Mob 1 was deleterious for conjugation even in the presence of MobI expressed *in trans*, indicating that the Mob 2 region could not complement the absence of the transfer functions of Mob 1. Further support for this idea was derived from an experiment in which the mobilization of the “*oriT*2” sequence was investigated in the presence of wt or the Δ*oriT* mutant R16a. As Δ*oriT* deletion eliminates *mobI* expression, which might also be required for “*oriT*2” mobilization (it was not clear at this stage), the “*oriT*2” sequence was inserted into a *mobI* expression vector supplying MobI protein. The resulting plasmid, pJKI1045, was mobilized by wt R16a at a frequency of 2.7 ± 1.7 × 10^−6^, while its mobilization by Δ*oriT* R16a was undetectable (<2.8 ± 1.0 × 10^−8^), suggesting that the transfer of “*oriT*2” depends on the conjugation of the helper plasmid but not directly on the presence of MobI.

The analyses of Mob 2 deletion mutants further strengthened our suspicion. The sequence in the Mob 2 region, which behaved like *oriT* when inserted into the p15A plasmid (provisionally called “*oriT*2”), was localized between the conserved divergent ORFs *R55_128* and *R55_196* with unknown functions (Fig. 1c). Before the generation of deletion mutations, the sequences of the homologous regions in R55, R16a and IP40a were compared, which revealed large interrupting insertions in this region in R16a and IP40a^30^. Both insertions (the 30-kb ARI_R16a_ and the 11.5-kb Tn*6333*) separated the “*oriT*2” sequence from the homologs of ORF *R55_196* without an apparent deleterious effect on the transfer of R16a and IP40a. Thus, based on the observation that the genetic context of “*oriT*2” and *R55_196* is not conserved in the three plasmids, we supposed that *R55_128* might be the corresponding accessory gene for the presumptive “*oriT*2” (as is *mobI* for *oriT* in the Mob 1 region) rather than *R55_196*. Deletions of the “*oriT*2” sequence or the homologs of ORF *R55_128* were generated in R16a and IP40a, and the mutants were tested for their conjugation activity. None of the deletions had a detectable effect (Supplementary Fig. 2), further supporting that this region does not carry *oriT* despite the previous observation that the sequence designated as “*oriT*2” could somehow be transferred if it was cloned into a non-mobilizable vector (Fig. 1c).

When comparing the incidence of co-transfer of the helper plasmid R55 and the p15A-based subclones of the Mob 1 and Mob 2 regions, a striking difference was observed. While the frequency of transconjugants that received R55 along with Mob 1 (*oriT*)-containing plasmids (pJKI1001, pJKI1006, pJKI1007 and pJKI1008) ranged between 25% and 61%, the Mob 2 (“*oriT*2”)-containing plasmids (pJKI972, pJKI984 or pJKI998) showed a prevalence of similar double transconjugants ranging from 99–100%. The same phenomenon was observed for pJKI1045 (*oriT*2^+^, *mobI* ^+^) transferred by wt R16a. This rate of co-transfer was unexpectedly high assuming the independent transfer of the helper and the tested plasmids. This result raised the possibility that the high frequency was due to cointegrate formation even in the *recA* ^−^ background of TG2 donor cells via homology-dependent recombination between the Mob 2 sequences present in both the helper and the Mob2-bearing plasmids. To test this hypothesis, PCRs amplifying the expected junctions of such cointegrates were performed using the transconjugant colonies bearing pJKI972 and pJKI998. However, no junction fragments could be amplified despite the presence of both R55 and p15A plasmids in the transconjugants.

**Figure 4:**
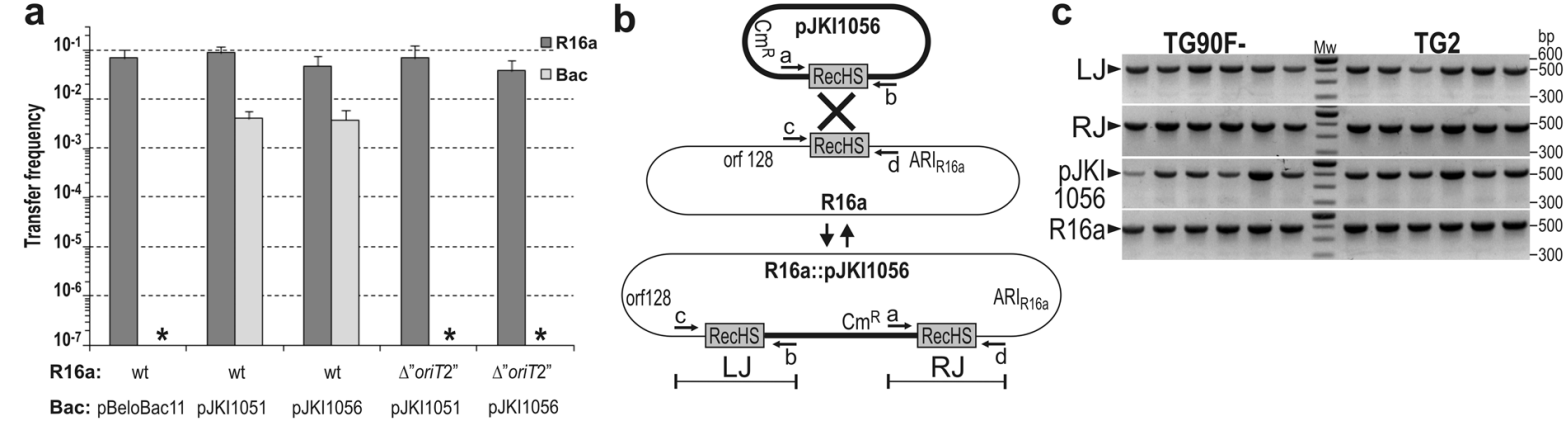
Transfer of RecHS-bearing plasmids through cointegrate formation with R16a helper plasmid. (**a**)The transfer frequency of pJKI1051 and pJKI1056 in the presence of the wt and the “Δ*oriT*2” R16a helper plasmids. The donor strain TG1Nal contained the helper plasmid along with the Bac-based RecHS-bearing plasmids pJKI1051 or pJKI1056. *E*. *coli* TG90F^−^ was used as the recipient strain. Asterisks indicate that transfer of pBeloBac11 vector by the wt R16a and the RecHS-bearing plasmids by the “Δ*oriT*2” R16a helper plasmid was undetectable (<3.0 × 10^−8^). (**b**) The schematic graph shows pJKI1056 (thick line), the wt R16a (thin line) and the expected cointegrate formed by recombination via the RecHS copies. ARI_R16a_ refers to the antibiotic resistance island in R16a inserted downstream of RecHS, orf128 denotes the homolog of ORF *R55_128* located upstream of RecHS and Cm^R^ indicates the position of the resistance gene in pJKI1056. Primers used for detection of the left (LJ) and right (RJ) junctions of R16a::pJKI1056 cointegrate and the free pJKI1056 and R16a plasmids are indicated. (**c**) Detection of the R16a::pJKI1056 cointegrate and the free parental plasmids by colony PCR in six independent transconjugant colonies obtained using TG90F^−^ (lanes 1–6) and *recA* ^−^ TG2 (lanes 8–13) recipients. To eliminate donor contamination, the transconjugant colonies were streaked onto LB + Tc + Km + Cm agar plates, and single colonies were selected as templates in the PCR tests. The primer pairs used and the length of the resulting amplicons were as follows: c–b for LJ (513 bp), a–d for RJ (516 bp), a-b for pJKI1056 (515 bp) and c,d for R16a (499 bp) (a, cat3; b, pUCfor21; c, R55_T1for; d, IP40/R16_T1rev). Mw: 100-bp ladder (Invitrogen). The full-length gel is presented in Supplementary Fig. 4.

Based on this negative result, we supposed that the transconjugant cells that had acquired a cointegrate of the 10–15-copy-number p15A plasmid and that the 170-kb IncA/C plasmid were not viable unless the cointegrate was resolved. Theoretically, such cells should have replicated the cointegrate until its copy number reached the standard copy number of the p15A replicon, which would mean the synthesis of ca. 2.3 Mb extra DNA (half of the chromosomal DNA) in each cell cycle. Therefore, it is more probable that those transconjugant cells formed colonies that escaped this load by resolving these “high copy” cointegrates. Thus, we expected the cointegrates to be more stable if their copy number did not exceed 1–2 per cell. To verify this hypothesis, the 350-bp and the shortest, 56-bp, transferable fragment of the Mob 2 region (corresponding to the inserts of pJKI972 and pJKI1053, respectively) were cloned into the single copy F plasmid derivative pBeloBac11. The resulting plasmids, pJKI1051 and pJKI1056, were introduced into TG1Nal/R16a and TG1Nal/R16a Δ“*oriT*2” cells, and their transfer frequency was measured using the F′-cured TG90 strain as a recipient. When wt R16a was applied as a helper, the transfer efficiency of both plasmids was ca. 1/10 that of the helper plasmid (Fig. 4a), independently of the length of the Mob 2 region present in the single copy vector as was observed previously for a series of p15A-based Mob 2 bearing plasmids (Fig. 1c). The resistance phenotype tested for >60 transconjugant colonies from four parallel experiments revealed a co-transfer rate of Bac-based pJKI1051 and pJKI1056 with R16a of 100%. Contrarily, no transconjugants were obtained with the R16a Δ“*oriT*2” helper, even though its transfer frequency was similar to that of wt R16a. Similar results were obtained when the *recA* ^−^ TG2 strain was used as the recipient strain in the same experimental setup, except that significantly lower transfer frequencies were detected for the Bac-based plasmids probably due to the presence of the incompatible F′ plasmid in the recipient strain (Supplementary Fig. 3). Colony PCR designed to detect the expected junctions of R16a::pJKI1056 cointegrates (Fig. 4b,c) confirmed that all the tested transconjugant colonies contained both the cointegrate and its resolution derivatives, free pJKI1056 and R16a. This result verified that the single-copy cointegrate was sufficiently stable to be maintained until the transconjugant cells formed colonies, but the reversed recombination process resolved the cointegrates at a detectable rate. The above results clearly indicate that the “*oriT*2” sequence is not a real *oriT* but contains a highly recombinogenic sequence renamed as RecHS (“Recombination Hot Spot”).

### RecHS is conserved in the IncA/C family and recognized by a plasmid-encoded recombinase

A comparison of the transferable and non-transferable fragments of the Mob 2 region indicated that the recombination activity depends on the intactness of the 14-bp imperfect inverse repeat (IR2) with 6-bp spacing (compare pJKI1053 and pJKI1054, Fig. 1c), while the 13-bp perfect inverse repeat with 1-bp spacing (IR1) located 27 bp upstream of IR2 seems dispensable for the recombination (compare pJKI998, pJKI999 and pJKI1053, Fig. 1c). The RecHS sequence was well conserved among the >150 sequenced IncA/C plasmids. Apart from the 14 family members that were missing this region, only XNC1_p and XNC2 carried a single A→G change in the right arm of IR2 (IR2R), and 28 of 138 members contained 1 or 3 divergent bases in the spacer region (Supplementary Fig. 5).

**Figure 5:**
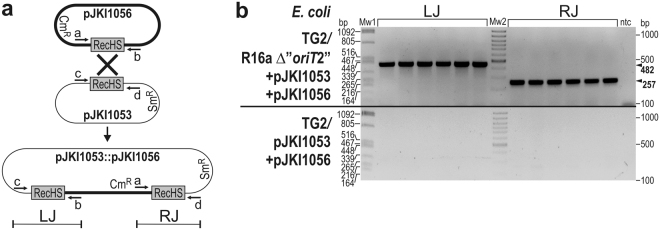
Detection of the cognate recombinase of RecHS. (**a**) The graph shows the Bac- (thick line) and p15A-based (thin line) RecHS-bearing plasmids and the expected cointegrate formed by recombination via RecHS copies. (**b**) Colony PCR was conducted for six independent transformant *E*. *coli* TG2 colonies carrying pJKI1053 and pJKI1056 with or without “Δ*oriT*2” R16a (the deletion mutant R16a lacking RecHS was used to avoid recombination with the other two plasmids). The left and right junctions of the pJKI1053::pJKI1056 cointegrates (LJ and RJ, respectively) could be amplified only in the presence of R16a as a 482-bp and a 257-bp fragment. The primers used were c–b for LJ and a–d for RJ (a, cat3; b, pUCfor21; c, pBRBgl; d, pBRPst). Mw1, lambda DNA digested with *Pst*I; Mw2, 100-bp ladder (Fermentas); ntc, nontemplate control.

The previous results ruled out the involvement of RecA in RecHS-recombination, and thus we were interested in whether the IncA/C plasmid or the host cell encode the cognate recombinase. The compatible plasmids pJKI1053 (p15A) and pJKI1056 (Bac) carrying the same RecHS sequence were introduced into TG2 and TG2/R16a Δ“*oriT*2” cells (R16a Δ“*oriT*2” lacks RecHS and was used to avoid recombination of the test plasmids with the IncA/C plasmid), and the transformant colonies were tested by PCR for the presence of pJKI1053::pJKI1056 cointegrates. Based on the detection of cointegrates only in the presence of R16a Δ“*oriT*2” (Fig. 5) it was confirmed that the IncA/C plasmid itself encodes the cognate recombinase.

## Discussion

IncA/C plasmids are of great importance due to their efficiency in the spread of multiresistance in *Enterobacteriaceae* and several other families of γ-Proteobacteria. Although the first IncA/C family plasmids were identified in the late 1960s^31, 32^, details of their basic biology have been studied only recently. Most genes of their conjugative transfer system have been identified based on their homology to other genes with known functions. Genetic analyses have been reported on the regulation of *tra* genes^19^ and on the genetic background of crosstalk between IncA/C plasmids and SGI1 genomic islands mobilized exclusively by these plasmids^19, 33, 34, 35^. Although most genes of the transfer apparatus have been identified, the cis-acting element of transfer initiation, the *oriT* region, has not yet been unambiguously defined. *OriT* was first localized between *traD* and *traJ* ^23^; however, this location was later disproved, and an *oriT* region was identified in the noncoding region upstream of *mobI*. Deletions generated in the predicted *oriT* sequence caused a ca. one order of magnitude drop in transfer frequency, but plasmid transfer was not abolished, which led to the suggestion that a second *oriT* may exist^20^.

Here, we report the more precise localization of the IncA/C *oriT* sequence and exclude the possibility of a second one. However, we identified a second region that could mimic the function of *oriT* in the experimental setup applied for the isolation of *oriT*. Using R55 fragment libraries, we identified two distinct regions of R55, Mob 1 and Mob 2, that rendered the non-mobile p15A plasmid mobilizable by an intact R55 helper plasmid, thus appearing to carry an *oriT*. In a similar test, the presence of an *oriT* in the ca. 11.6-kb surroundings of the relaxase gene, *traI*, was excluded.

Mob 1 of R55 was shown to be homologous to the region in which *oriT* of another IncA/C plasmid, pVCR94, was recognized^20^. Based on the transfer frequencies of the sequentially shortened subclones of the Mob 1 region, *oriT* was identified in the 139–323 bp region of R55 immediately preceding the *mobI* gene (Fig. 1a,b). Unlike the previously reported ΔoriT1_94 and ΔoriT2_94 deletions (corresponding to 39–203 bp and 170671–203 bp segments of R55, respectively)^20^ in pVCR94ΔX, the Δ*oriT* deletions removing the 129-bp and 115-bp stretches upstream of the *mobI* gene in the related IncA/C plasmids R16a and IP40a completely abolished plasmid transfer and confirmed that the most important parts of *oriT* are immediately adjacent to *mobI*.

Sequence analysis of the 139–323 bp region revealed a strong bias in GC content. In contrast to the 53% GC of the whole R55 and 51% of its conserved backbone sequence (excluding ARI-B and ARI_R55_/Tn*6187* ^30^), *oriT* was found to contain only 37% GC. Furthermore, a complex array of short perfect and imperfect inverted and direct repeats (Fig. 1b) could be found, as is also characteristic of several other *oriT* loci^5, 6, 8, 9, 10^. Further shortening of the 139–323 bp region had a negative effect on the transfer rates. Removal of the upstream segment, including IR1 and IR2, caused a minor reduction, indicating that this region contained sequences with some non-essential roles in the transfer. This result is supported by the ca. ten-fold decrease in the transfer rate observed with ΔoriT1_94 and ΔoriT2_94 deletion mutants of pVCR94ΔX^20^, both of which removed distal parts of upstream sequences of *oriT*, including IR1 and IR2. Because the absence of the 97–139 bp region had no detectable impact on the transfer rate (compare pJKI974 and pJKI985 in Fig. 1a,b), in this side of *oriT*, the effective sequence(s) must be located between 139–208 bp, near or even overlapping the IR1 or IR2 motifs. Sequential shortening of the *oriT* region from the other side (adjacent to *mobI*) exhibited a gradual effect. Deletion of the 323–353 bp region with IR6 had no impact, while removal of one copy of the overlapping 14-bp DRs caused a slight decrease in the transfer rate. However, the removal of both DRs, which also destroys IR5, eliminated the transfer, suggesting that this region encompassed the most important elements of *oriT* (such as the nic-site and indispensable binding sites). Interestingly, simultaneous deletion of the IR1/IR2 region and the 14-bp DR2 (pJKI1006) had a cumulative effect as it led to a 2 log drop in transfer frequency, similar to the deletion of the region from IR1 to IR3 (pJKI1008). Thus, we concluded that the core of *oriT* is located in the 227–300 bp fragment (pJKI1007), including IR4, IR5 and at least one copy of the 14-bp DR, but the fully functional *oriT* is covered by the 139–323 bp region.

The comparative analysis of sequences homologous to the *oriT* region of R55 also supports this conclusion. To date, more than 150 sequenced IncA/C family members can be found in public databases (Supplementary Table 2). Even though 5 of 152 plasmids lack *oriT* due to large deletions spanning the whole Mob 1 region, including the *mobI* gene, this region is highly conserved. Fifty-seven of the remaining 147 plasmids show some sequence divergence in comparison to R55, while 89 are identical to it (Supplementary Fig. 6). The *oriT* locus of the six most divergent plasmids begins at the 169/170 bp position of R55. This group includes two plasmids that have been reported to undergo conjugal transfer: pRA1^36, 23^, the only sequenced IncA/C_1_ plasmid, and pKHM-1^37^, which cannot be unambiguously classified into Type 1 or Type 2 clusters of the IncA/C_2_ lineage^30^. The self-transfer capability of these plasmids confirms again that the missing region, including IR1, is not necessary. In the 170–323 bp region of R55, there are three positions in which the other plasmids contain divergent bases; however, these positions occur in the spacing of IRs (Supplementary Fig. 6, Fig. 1b). The only striking variance in the core *oriT* region can be seen in R16a, where three rather than two copies of the overlapping 14-bp DRs were found^30^. Comparison of the trans mobilization of R16a and R55 Mob 1 regions by both plasmids showed that this additional repeat had no significant effect. Based on this compilation of sequences, *oriT* of the IncA/C plasmids could be determined as the 170–323 bp of R55 containing IRs from IR2 to IR5 and the 14-bp DRs; however, the functions of these repeats remain to be established. Although there have been many efforts to identify the *nic* site in *oriT*, both biochemical (primer extension) and *in vivo* (interrupted mating) approaches have failed to provide positive results, and thus further *in vitro* assays performed with purified proteins might help to solve this problem.

The *oriT* is located immediately upstream of *mobI*, a highly conserved gene (Supplementary Table 2) that is necessary for plasmid transfer^20^. *MobI* was identified due to its analogous genetic context to *mobI* of SXT^21^, although its function has not yet been determined. Swiss-Model and Phyre2 modeling of the MobI protein structure have predicted some homology in the C terminus with the DNA-binding domain of the EIN3 transcription factor of *Arabidopsis thaliana*, raising the possibility that MobI has DNA binding activity and might have similar functions in transfer to *oriT*-binding auxiliary proteins as TraJ of RP4^12^, TraY of F factor^38^, TrwA of R388^14^ or MobC of RA3^39^. Although the exact function and mode of action of MobI are not yet clear, we demonstrated that it is a plasmid-specific transfer factor that is not required for the trans mobilization of SGI1.

β-galactosidase assay showed that the upstream region of *mobI* contained a relatively strong promoter, which was identified by a primer extension experiment. The most abundant transcript started at the 229 bp position of R55 which defined a promoter with a −35 box (TTGACA) that aligned perfectly with the consensus σ^70^ promoter box followed by a less ideal −10 box (TAGCCT) with 16-bp spacing. Interestingly, four additional secondary TSSs were also detected closer to the start codon (Fig. 3). All the TSSs and the corresponding promoters were located in the most important part of *oriT*. Similar overlap between *oriT* and the promoter of adjacent genes has been found in several plasmids from other families such as RP4^7^, R100^40^, RA3^39^, pMV158^41^ and pIP501^42^. This context in IncA/C *oriT* raises the possibility of crosstalk between *mobI* transcription initiation and the formation of the initiation complex of conjugal transfer. Furthermore, binding of MobI to *oriT* might lead to the autoregulation of *mobI* transcription.

The method by which the Mob 1 region including *oriT* was entrapped provided a second region, Mob 2, that also appeared to be mobilizable. Further analyses, however, ruled out the possibility that Mob 2 carried a second *oriT*. Conjugation tests for the gradually shortened Mob 2 region fragments inserted into a p15A-based vector led to the identification of a 56-bp region, RecHS, that appeared to be fully active in the transfer by a helper plasmid, but the deletion of this region from R16a or IP40a had no impact on their conjugation frequencies. By contrast, the deletion of *oriT* in the Mob 1 region abolished conjugation, which excluded the presence of a second *oriT* either in Mob 2 or in any other regions of the plasmids. The nearly 100% co-transfer of Mob 2 bearing p15A- or Bac-based plasmids with the helper plasmid and the complete termination of their transfer when the helper was unable to conjugate (Δ*oriT*) or lacked the RecHS (Δ“*oriT*2”) indicated that transfer of the Mob 2 region undoubtedly occurred via cointegrate formation with the helper plasmid. We have shown that cointegrates are formed via a RecA-independent recombination that occurs between two RecHS sequences. The presence of the IncA/C plasmid is necessary for the recombination, indicating that the plasmid itself encodes the cognate recombinase. It was also shown that this enzyme is able to carry out the recombination process in both directions i.e. the formation or the resolution of cointegrates.

The RecHS region contains a 14-bp imperfect inverted repeat with 6-bp spacing (IR2). Neither the Mob 2 fragments lacking IR2 (pJKI965, Fig. 1c) nor those carrying only one copy of the IRs (pJKI997, pJKI999 and pJKI1054, Fig. 1c) could be transferred, implying a key role for this motif in recombination-dependent cointegrate formation. The structure of IR2 clearly resembles the recombination sites of several site-specific recombination systems that are involved in the dimer resolution of plasmids, such as *rfsF* of F factor^43^, *FRT* of 2 μm plasmid^44^ of yeast or *loxP* of P1 phage^45^. All these sites consist of 10–14-bp imperfect inverted repeats with a central 6–8-bp spacer in which strand cleavage and rejoining are carried out by the cognate recombinases. The chromosomal and several plasmid-borne dimer resolution systems (XerCD-*dif/cer*) have similar functions, but their recombination sites, *dif* and *cer*, are not composed of inverted repeats^46^. The *res* loci of another group of cointegrate resolution systems occurring in Tn*3*-family replicative transposons also contain inverse repeats with 2–7-bp spacers, although *res* sites generally consist of three similar copies of IRs (*res*I, II and III) with different spacing lengths between the repeated motifs^47^. Although the cognate recombinase of RecHS has not yet been identified, we believe that IR2 in the RecHS sequence acts as a recombination site in a site-specific system similar to the *rfsF*-D protein system of F plasmid^48^. High conservation of the RecHS sequence in the IncA/C family (Supplementary Fig. 5) suggests that it has an important function. Based on the analogy to several dimer resolution systems, RecHS might have a role in plasmid stability. However, 14 of 152 IncA/C plasmids lack the RecHS region (Supplementary Table 2), indicating that this system is not necessary for stable maintenance, presumably due to other stabilizing functions. The detailed analysis of this recombination system is currently underway.

## Methods

### DNA and microbial techniques

Relevant features of the bacterial strains and plasmids used in this study are listed in Table 2. The detailed methodology of the plasmid constructions is described in Supplementary Methods. Standard molecular biology procedures were carried out according to^49^. Test PCRs were performed as previously described^50^ using Dream Taq polymerase (Thermo Fisher Scientific). All cloned amplicons were amplified with Phusion polymerase (Thermo Fisher Scientific) and sequenced on an ABI Prism 3100 Genetic Analyzer (Perkin Elmer) after cloning. Oligonucleotide primers are listed in Supplementary Table 3. Primers for gene KOs were designed according to the published sequences of R55 (GenBank: JQ010984), R16a (GenBank: KX156773) and IP40a (GenBank: KX156772). Bacterial strains were routinely grown at 37 °C in LB supplemented with the appropriate antibiotics used at a final concentration as follows: ampicillin (Ap) 150 μg/ml, chloramphenicol (Cm) 20 μg/ml, kanamycin (Km) 30 μg/ml, spectinomycin (Sp) 50 μg/ml, streptomycin (Sm) 50 μg/ml, nalidixic acid (Nal) 20 μg/ml, gentamicin (Gm) 25 μg/ml, tetracycline (Tc) 10 μg/ml. For maintaining and curing the plasmids with the temperature-sensitive pSC101 replication system, 30 and 42 °C incubation temperatures were applied, respectively. Standard conjugation assays were carried out in 4–6 replicates and 2–6 independent experiments as previously described^50^. The transfer frequencies are given as the ratio of transconjugant and recipient titers, if other is not indicated. The β-galactosidase assay was performed in 4 replicates according to^51^ except that the cultures were grown in LB at 37 °C to an OD_600_ ~0.3 and diluted in at a 1:9 ratio with Z buffer.

**Table 2:** Relevant features of bacterial strains and plasmids used in this study.

Strains and Plasmids	Genotype or relevant features^a^	References
*E*. *coli*		
TG1	*supE hsd*Δ5 *thi* Δ(*lac-proAB*) F’[*traD36 proAB*+ *lacIq lacZ*ΔM15]	56
TG1Nal	Nal^R^ derivative of TG1	50
TG2	*supE hsd*Δ5 *thi* Δ(*lac-proAB*)∆(*srl-recA*)306::Tn10(Tc^R^) F’[*traD36 proAB*+ *lacIq lacZ*ΔM15]	49
TG90	*pcn B80 zad*::Tn10 (Tc^R^) derivative of TG1	57
TG90Nal	Nal^R^ derivative of TG90	50
TG90F-	F′-cured derivative of TG90	30
TG1Nal/R55	TG1Nal strain containing R55, Nal^R^, Ap^R^, Cm^R^, Flo^R^, Su^R^, Km^R^, Gm^R^	33
TG1Nal/R16a	TG1Nal strain containing R16a, Nal^R^, Ap^R^, Km^R^, Sul^R^	30
TG1Nal::SGI1-C	TG1Nal strain containing SGI1-C variant integrated into *E*. *coli thdF*, Nal^R^, Sm^R^, Sp^R^	33
TG1Nal/IP40a	TG1Nal strain containing IP40a, Nal^R^, Ap^R^, Km^R^, Sul^R^	30
TG2/R55	TG2 containing R55, Tc^R^, Ap^R^, Cm^R^, Flo^R^, Km^R^, Gm^R^, Sul^R^	this work
TG2/R16a	TG2 strain containing R16a, Nal^R^, Ap^R^, Km^R^, Sul^R^	this work
Plasmids		
R55	IncC Type2, tra+, Ap^R^, Km^R^, Cm^R^, Flo^R^, Gm^R^, Sul^R^	32
R16a	IncC Type1, tra+, Ap^R^, Km^R^, Sul^R^	32
IP40a	IncC Type1, tra+, Ap^R^, Km^R^, Sul^R^	32
pBeloBac11	F plasmid based Cm^R^ cloning vector	NEB
pBluescript II-SK	pMB1-based Ap^R^ cloning vector	58
pEMBL19	pMB1-based Ap^R^ cloning vector	59
pKD3	Cm^R^, Ap^R^ R6Kγ-based PCR template plasmid for one-step recombination gene-KO	26
pKD46	Ap^R^ ara-inducible expression vector of λ Red recombinase with temperature-sensitive pSC101 replication system	26
pJKI648	Ap^S^, Gm^R^ derivative of pKD46, the Gm^R^ cassette is inserted into the Ap^R^ gene	33
pJKI708	Sm^R^ derivative of the p15A-based cloning vector, pJKI88^60^ deriving from pACYC177^29^	this work
pJKI854	9698 bp *Hpa*I fragment of R16a carrying *traI* KO::Cm^R^ cloned into pJKI708	this work
pJKI962	118709–119458 bp *Sac*I fragment of R55 cloned in pJKI708 (Mob 2 region)	this work
pJKI963	169567–170810/1–1587 bp *Sac*I fragment of R55 cloned in pJKI708 (Mob 1 region)	this work
pJKI964	168440–170810/1–353 bp *Bst*YI fragment of R55 cloned in pJKI708 (Mob 1 region)	this work
pJKI965	118709–118981 bp fragment of R55, *Bgl*II-*Bam*HI deletion derivative of pJKI962 (Mob 2 region)	this work
pJKI966	118978–119458 bp *Bgl*II-*Sac*I fragment of R55 cloned in pJKI708 (Mob 2 region)	this work
pJKI967	169567–170810/1–353 bp fragment of R55, *Bgl*II-*Bam*HI deletion derivative of pJKI963 (Mob 1 region)	this work
pJKI968	169567–170501 bp fragment of R55, *Hin*cII deletion derivative of pJKI963 (Mob 1 region)	this work
pJKI969	170501–170810/1–353 bp fragment of R55, *Hin*cII deletion derivative of pJKI964 (Mob 1 region)	this work
pJKI972	118978–119327 bp fragment of R55 cloned in pJKI708 (Mob 2 region)	this work
pJKI973	170501–170810/1–136 bp fragment of R55, *Eco*RI-*Nde*I deletion derivative of pJKI969 (Mob 1 region)	this work
pJKI974	139–353 bp fragment of R55, *Nde*I-*Pst*I deletion derivative of pJKI969 (Mob 1 region)	this work
pJKI981	119000–119327 bp fragment of R55 cloned in pJKI708 (Mob 2 region)	this work
pJKI982	119000–119224 bp fragment of R55 cloned in pJKI708 (Mob 2 region)	this work
pJKI983	119065–119327 bp fragment of R55 cloned in pJKI708 (Mob 2 region)	this work
pJKI984	119065–119224 bp fragment of R55 cloned in pJKI708 (Mob 2 region)	this work
pJKI985	97–353 bp fragment of R55 cloned in pJKI708 (Mob 1 region)	this work
pJKI986	97–323 bp fragment of R55 cloned in pJKI708 (Mob 1 region)	this work
pJKI987	97–280 bp fragment of R55 cloned in pJKI708 (Mob 1 region)	this work
pJKI997	119065–119190 bp fragment of R55 cloned in pJKI708 (Mob 2 region)	this work
pJKI998	119107–119224 bp fragment of R55 cloned in pJKI708 (Mob 2 region)	this work
pJKI999	119107–119190 bp fragment of R55 cloned in pJKI708 (Mob 2 region)	this work
pJKI1000	177–323 bp fragment of R55 cloned in pJKI708 (Mob 1 region)	this work
pJKI1001	208–323 bp fragment of R55 cloned in pJKI708 (Mob 1 region)	this work
pJKI1002	97–300 bp fragment of R55 cloned in pJKI708 (Mob 1 region)	this work
pJKI1006	208–300 bp fragment of R55 cloned in pJKI708 (Mob 1 region)	this work
pJKI1007	227–300 bp fragment of R55 cloned in pJKI708 (Mob 1 region)	this work
pJKI1008	227–323 bp fragment of R55 cloned in pJKI708 (Mob 1 region)	this work
pJKI1011	170501–170810/1–863 bp fragment of R55 cloned in pJKI708 (Mob 1 region and *mob*I gene)	this work
pJKI1012	97–410 bp fragment of R16a cloned in pJKI708 (Mob 1 region)	this work
pJKI1021	Sm^R^ p15A-based pJKI391^33^-derived expression vector containing *mobI* under the control of P_*tac*_	this work
pJKI1045	118978–119326 bp Mob 2 region of R55 inserted into the *mob*I expressing plasmid pJKI1021	this work
pJKI1051	118978–119326 bp fragment of R55 cloned in pBeloBac11 (Mob 2 region)	this work
pJKI1053	119169–119224 bp fragment of R55 cloned in pJKI708 (Mob 2 region)	this work
pJKI1054	119191–119224 bp fragment of R55 cloned in pJKI708 (Mob 2 region)	this work
pJKI1056	119169–119224 bp fragment of R55 in cloned pBeloBac11 (Mob 2 region)	this work
pMSZ952	pJKI990^33^-derivative β-galactosidase tester plasmid containing the non-coding upstream region of *mobI* (170632–170810/1–321 bp) fused to the promoterless *lacZ* gene.	this work

### Isolation of the Mob regions of R55

R55 libraries L1-L9 were generated by digestion of ca. 200 ng of R55 plasmid DNA purified with the Qiagen Plasmid Maxi kit. Plasmid DNA was cut with *Bam*HI-*Bgl*II (L1); *Bst*YI (L2); *Eco*RI-*Mfe*I (L3); *Apo*I (L4); *Sac*I (L5); *Hinc*II (L6); *Stu*I (L7); *Taq*I (L8); and *Sau*3AI (L9). Fragments were ligated into the *Bam*HI (L1, L2, L9), *Eco*RI (L3, L4), *Sac*I (L5), *Hinc*II (L6, L7) or *Acc*I (L8) sites of pJKI708. The nine libraries were transformed into TG2/R55 cells. After heat shock at 42 °C, the transformed cultures were incubated for 2 h at 37 °C in 1 ml LB medium, followed by addition of 1 ml LB+Sm+Sp broth and growth of the cultures O/N at 37 °C. The O/N cultures were centrifuged, washed with 0.9% NaCl solution and used as donors in crosses with 200 μl O/N cultures of the TG1Nal recipient strain as previously described^50^. After conjugation, the donor and recipient titers varied between 5.0 × 10^6^ − 1.2 × 10^8^ and 1.0−2.6 × 10^10^ CFU/ml, respectively. Transconjugants selected on LB + Nal + Sm + Sp plates were obtained only from libraries L1 (60 CFU/ml), L2 (30 CFU/ml) and L5 (4.0 × 10^4^ CFU/ml). To ensure the isolation of all mobilizable regions, the whole experiment was repeated twice, which provided similar results.

### One-step gene KO experiments

Gene KO experiments and elimination of the Cm^R^ marker gene from the KO alleles were conducted according to the one-step recombination method^26^ using the λ Red recombinase producer plasmid pJKI648^33^, a Gm^R^ derivative of pKD46. As R55 has multiple resistance genes (*floR*, *cat*, *aadB*, *oxa21*, *qacE*Δ*1*, *sulI*, *mer*), this plasmid was not applicable for one-step gene KO, and thus we used its close relatives, R16a and IP40a, to delete the *oriT*, *RecHS*, *traI*, *mobI*, and *orf128* genes. The KO fragments were amplified from the pKD3 template plasmid using primers R55_T2delfor – R55_T2delrev, R55_T1delfor – IP40/R16_T1delrev, deltraIR55for – deltraIR55rev, R55_001delstart – R55_001delstop and R55_128delfor – R55_128delrev, respectively.

### Primer extension analysis

Total RNA was isolated from *E*. *coli* strain TG1 containing the plasmid pMSZ952 carrying the non-coding upstream region of *mobI* followed by the promoterless *lacZ* gene, or pJKI990 as a negative control. RNA isolation and primer extension were carried out as previously described^34^. Overnight cultures were diluted 1000× and grown at 37 °C to an OD_600_ of 0.6 in 10 ml LB without antibiotics. Cells were harvested from 2.5 ml cultures and frozen in liquid nitrogen. Next, 750 μl of lysis buffer (0.2 M Na-acetate pH 5.2, 1% SDS 10 mM EDTA) was added to the frozen pellet. After vortexing, the mixture was boiled for 2–3 min and then vortexed again for 2 min. Lysates were centrifuged (20 min, 16000 rcf, 4 °C), and the supernatants were extracted with 750 μl phenol and centrifuged again (10 min, 16000 rcf, 4 °C). Extraction was repeated with 720 μl phenol: chloroform (1:1) followed by 360 μl chloroform. RNA was precipitated by adding 200 μl 10 M LiCl to 600 μl supernatant followed by incubation on ice (1 hour) and centrifugation (10 min, 16000 rcf, 4 °C). The pellet was first washed with 2.5 M LiCl and then with 70% ethanol, centrifuged, air-dried and dissolved in 50 μl RNase-free water at 50 °C. Then, 20 μl (~10 μg) of total RNA was digested with 50 units of RNase-free DNaseI (Qiagen) in a final volume of 50 μl (10 min, 37 °C), and the enzyme was subsequently inactivated (10 min, 65 °C). The reaction mixture was subjected to phenol-chloroform extraction, followed by two washes with 70% ethanol and resuspension in 20 μl RNase-free water after drying. The RNA concentration was set at 0.5 μg/μl.

The primer extension reaction was performed using the RevertAid H Minus first-strand cDNA synthesis kit (Fermentas). Ten pmoles of pUCfor21 primer was labeled in a 10-μl volume with 10 units of polynucleotide kinase (Fermentas) using 50 μCi [γ-^32^P] dATP (45 min, 37 °C). Subsequently, the enzyme was inactivated for 10 min at 68 °C. Approximately 5 μg of total RNA and 2 pmoles of^32^P-labeled primer were mixed, heated to 70 °C for 5 min, and then allowed to anneal at 37 °C for 5 min. The extension reactions were performed in RT buffer (50 mM Tris-HCl [pH 8.3], 50 mM KCl, 4 mM MgCl_2_ 10 mM DTT) with 1 mM dNTP and 20 units RiboLock ribonuclease inhibitor in a total volume of 20 μl (42 °C, 60 min) using 200 units of reverse transcriptase. The extension products were precipitated with ethanol, resuspended in 3 μl DEPC-treated water, and combined with 2 μl sequencing loading solution. The sequence ladder for the tester plasmids was generated with primer pUCfor 21 and pMSZ952 template plasmid using a Sequenase version 2.0 DNA sequencing kit (USB) according to the manufacturer’s protocol. The products of each reaction were electrophoresed on a 6% denaturing polyacrylamide gel at 1800 V. The gel was exposed to a storage phosphor screen and scanned on a Storm 840 PhosphorImager (Amersham Biosciences).

### Bioinformatics

Sequence alignments were generated using the MultAlin interface^52^. Protein structure modeling for MobI was conducted using Swiss-Model^53^ and Phyre2^54^. All homology searches were performed with the NCBI BLAST server. IncA/C family plasmids were identified via a nucleotide megaBLAST search in GenBank with the *repA* gene of R55 as the query sequence. Plasmids having a *repA* homolog with ≥98% alignment length coverage and ≥80% identity for a *repA* gene of R55, and carrying the large parts of the conserved backbone of R55 were considered as IncA/C plasmid (Supplementary Table 2). The potential secondary structure of *oriT* was generated using the mFold webserver^55^.

### Data availability

All data generated or analyzed during this study are included in this published article (and its Supplementary Information files).

## Electronic supplementary material

Identification of oriT and a recombination hot spot in the IncA/C plasmid backbone

Da taset 1
