# Supplementary material for: Identification of oriT and a recombination hot spot in the IncA/C plasmid backbone
Source: Sci Rep. 2017 Sep 6;7:10595. doi: 10.1038/s41598-017-11097-0 (PMC5587640; doi:10.1038/s41598-017-11097-0)
Supplement: Supplementary file 1 — Identification of oriT and a recombination hot spot in the IncA/C plasmid backbone [file 41598_2017_11097_MOESM1_ESM.pdf]

Supplementary information

Identification of *oriT* and a recombination hot spot in the IncA/C plasmid backbone

Anna Hegyi, Mónika Szabó, Ferenc Olasz, János Kiss

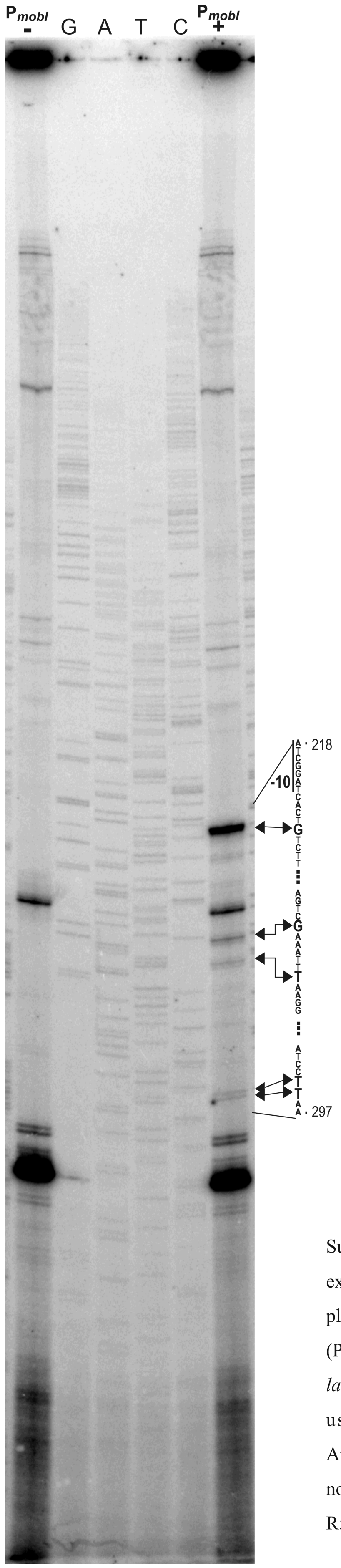

Supplementary Fig. 1. Determination of *mobI* TSS. The primer extension reaction was performed using the P_{mobI}::*lacZ* fusion plasmid pMSZ952 (P_{mobI}+) and pJKI990 as a negative control (P_{mobI}-) with primer pUCfor21 annealing near the start codon of *lac*Z. Lanes G, A, T, C: Sanger sequencing reactions obtained using pUCfor21 and pMSZ952 template DNA. Arrowheadsdenote the bases corresponding to the TSSs on the non-transcribed strand. The coordinates refer to the published R55 sequence (JQ010984).

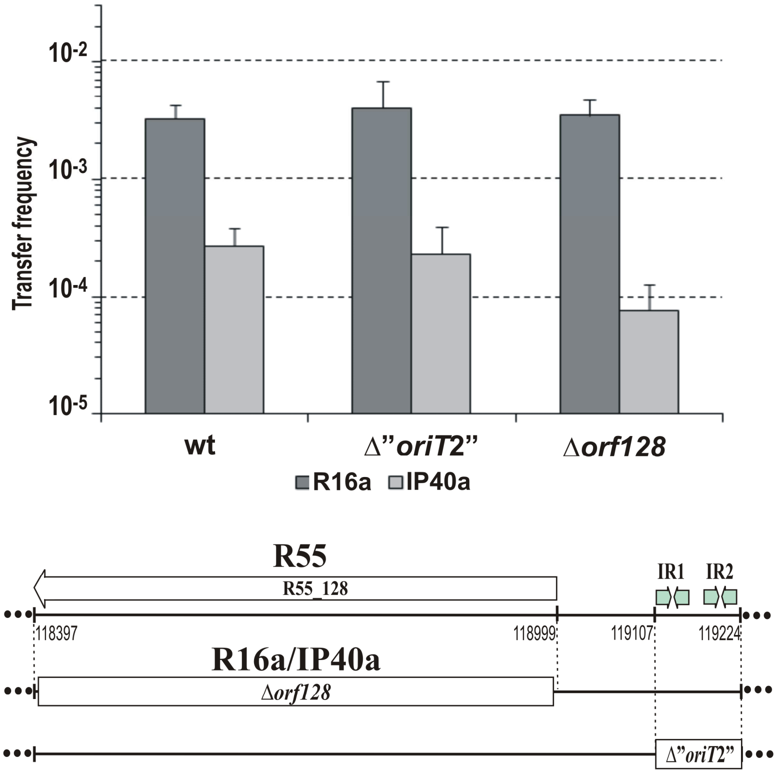

Supplementary Fig. 2. The effect of deletion mutations in the Mob 2 region of R16a and IP40a on plasmid transfer. The bars represent the mean values of five independent experiments. The schematic map of the Mob 2 region of R55 is shown below the graph. The same deletions (open boxes) were generated in R16a and IP40a. Symbols are indicated as in Fig. 1.

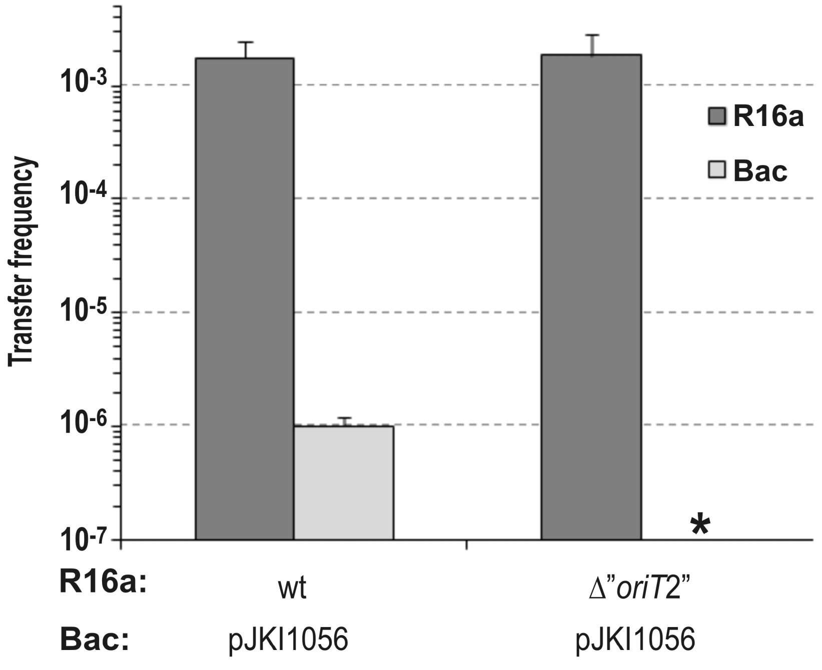

Supplementary Fig. 3. Transfer frequency of the RecHS-bearing Bac-based plasmid, pJKI1056, and the wt or "Δ*oriT*2" R16a helper plasmid. Bars represent the mean values of six independent conjugation experiments using the TG1Nal donor and the *recA*$^-$ TG2 recipient. Asterisk indicates that the transfer of pJKI1056 by "Δ*oriT*2" R16a helper was below the detection limit ($<2.8\times10^{-8}$).

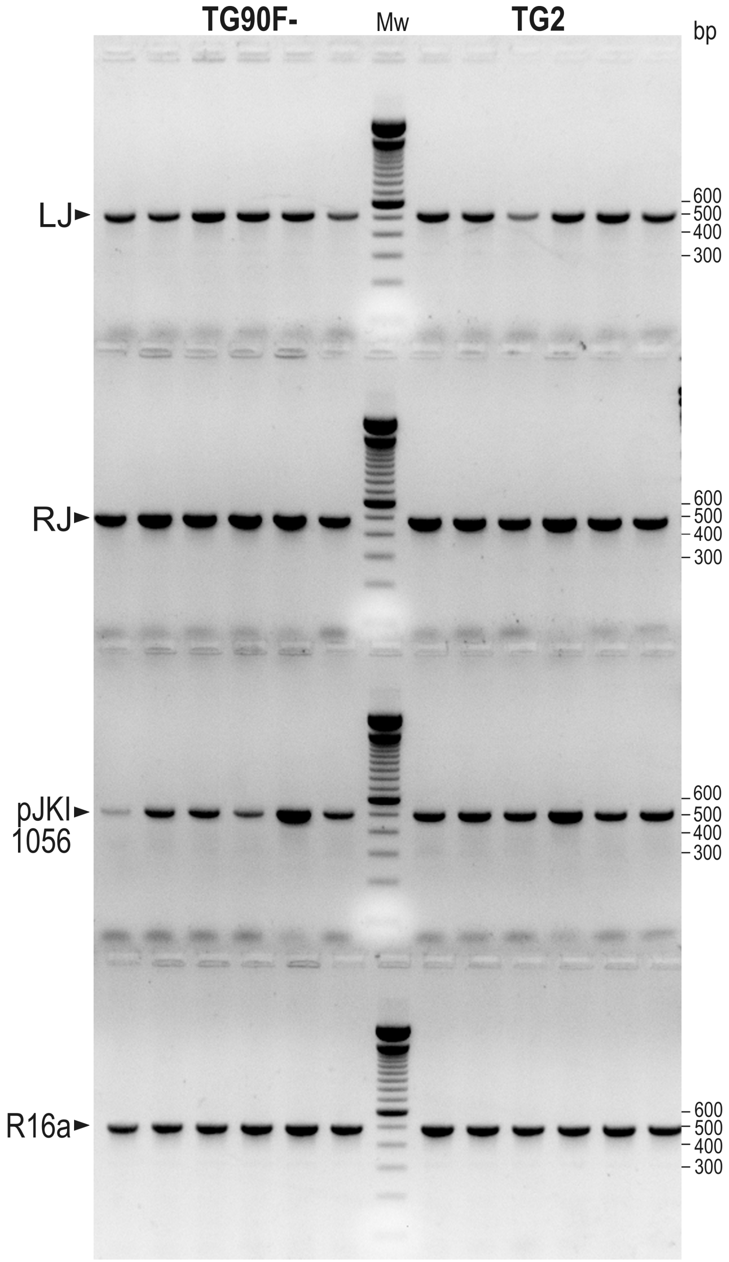

Supplementary Fig. 4. Detection of the left (LJ) and right (RJ) junctions of the R16a::pJKI1056 cointegrates and the free parental plasmids. The amplicons obtained by colony PCRs from six independent transconjugant colonies from TG90F^{-} (lanes 1-6) and the *recA*$^{-}$ TG2 (lanes 8-13) recipients are indicated.

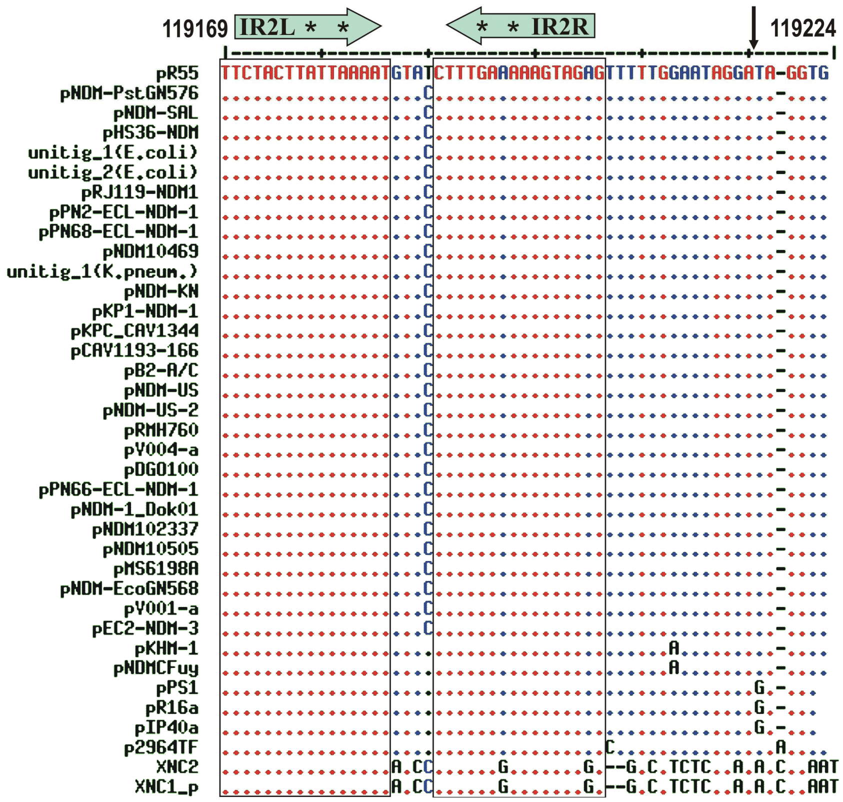

Supplementary Fig. 5. Comparison of RecHS sequences of IncA/C family plasmids. The alignment shows the sequence divergence in the RecHS regions corresponding to the 119190-119224 bp segment of R55 (insert in pJKI1056). Fourteen of 152 plasmids lacking the RecHS region are not shown (for the entire list of 152 IncA/C plasmids sequenced to date, see Supplementary Table 2). The 102 plasmids identical to R55 in the RecHS region are represented by the single R55 sequence, and only 36 divergent sequences are shown. Dots represent identity with R55. The 14-bp inverted repeats are denoted by arrows above the R55 sequence. Asterisks indicate mismatches of the IRs, and the vertical arrow points to the insertion site of Tn*6333* in IP40a and the Tn*6333*-based ARIs in R16a and pPS1, respectively. The most conserved regions are indicated by boxes.

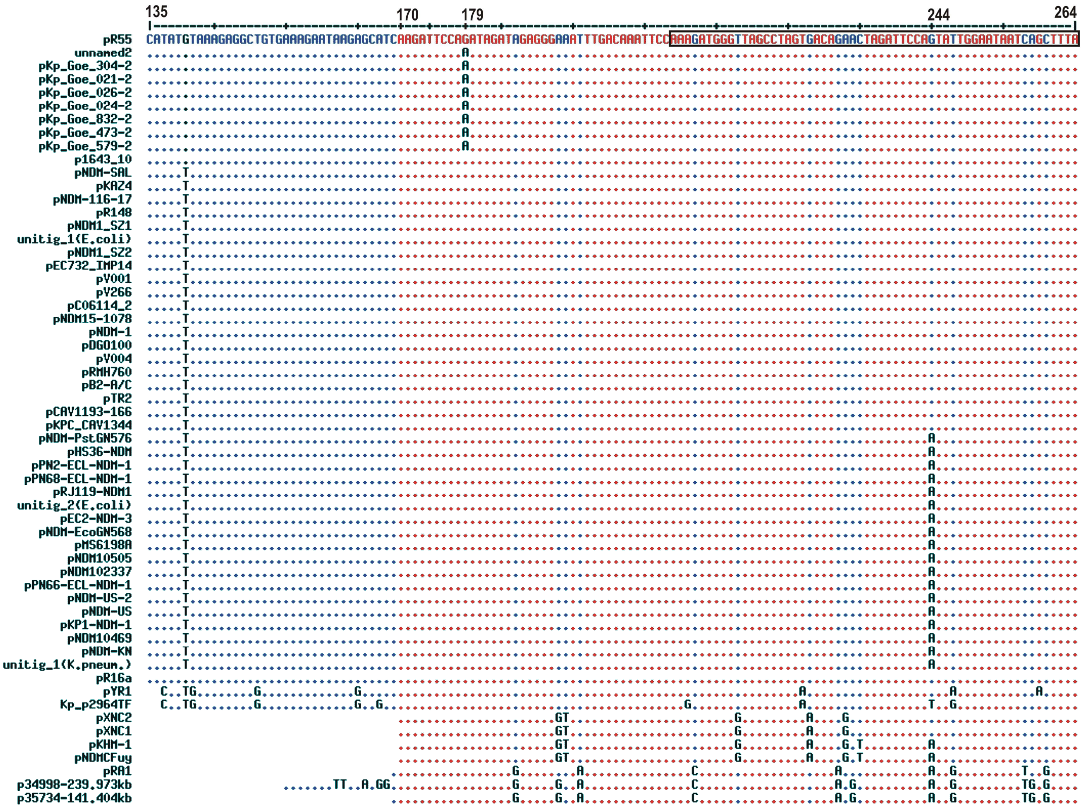

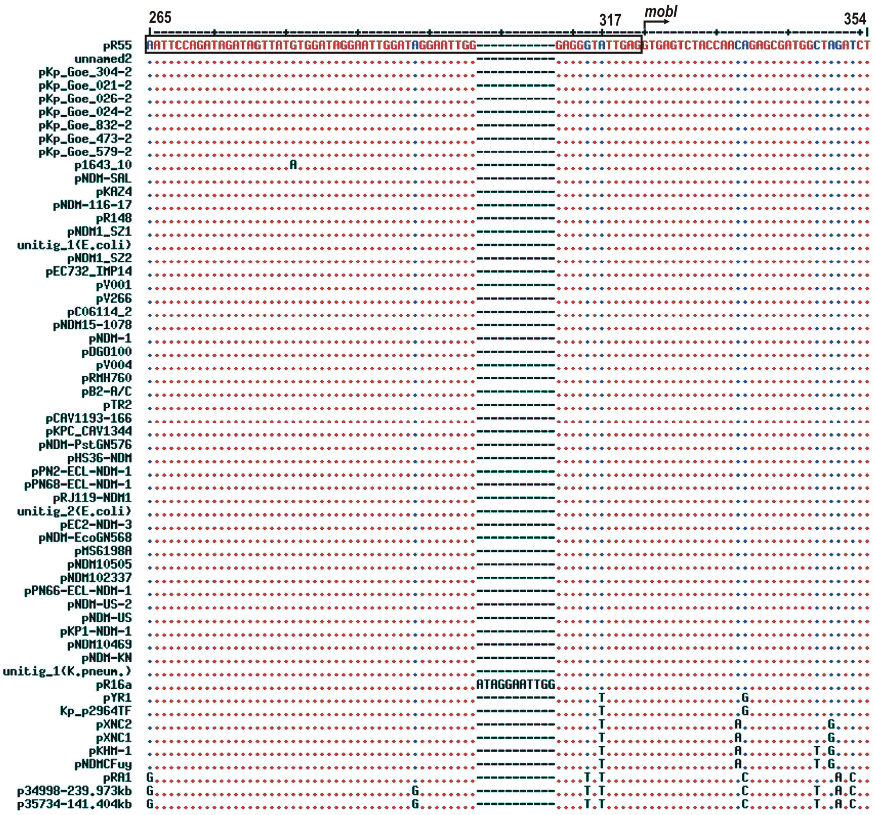

Supplementary Fig. 6. Comparison of Mob 1 regions of the IncA/C family plasmids. The alignment shows the sequence divergence in Mob 1 compared to the 135-354 bp sequence of R55 (corresponding to the insert in pJKI974 carrying the fully active *oriT*). Five of 152 plasmid lacking the Mob 1 region and *mobI* gene are not shown (for the entire list of IncA/C plasmids, see Supplementary Table 2). The 90 plasmids identical to R55 in this region are represented by the single R55 sequence at the top and the 57 divergent sequences are shown below. Dots represent identity with R55. The core *oriT* region (deleted in Δ*oriT* R16a and IP40a mutants) are boxed. Coordinates are shown according to R55 sequence.

Supplementary Table 1. Mobilization of the R16a and R55 *oriT* regions by the two IncA/C plasmids.

Helper	p15A::*oriT* plasmid	Rel. frequency of transconjugants.[a]
R16a	pJKI1012 (R16a *oriT*)	$1,2\times10^{-2}$
	pJKI986 (R55 *oriT*)	$5,5\times10^{-2}$
R55	pJKI1012 (R16a *oriT*)	$4,7\times10^{-3}$
	pJKI986 (R55 *oriT*)	$2,6\times10^{-3}$

[a] Data represent means of three replicates. Relative transfer frequencies of the *oriT*-containing p15A plasmids were calculated as the ratio of transfer frequencies of p15A and helper plasmids.

Supplementary Table 3. Oligonucleotide primers used.

Name	Sequence (5’→3’)	References
R55_T1for	ttgaattcgcttcgcatccttgggagctg	this work
R55_T1for1	ttgaattcattatctctcgcacctgtacatg	this work
R55_T1for2	ttgaattccagcgagacatcttatacc	this work
R55_T1for3	ttgaattcattaccgtattaccgtaattatg	this work
R55_T1for4	aagaattctacttattaaaatgtatctttg	this work
R55_T1rev	ttctgcagcacctatcctattccaaaaactc	this work
R55_T1rev2	ttctgcagatacattttaataagtagaacaacag	this work
recHSfor	aattcttgaaaaagtagagtttttggaataggactgca	this work
recHSrev	gtcctattccaaaaactctactttttcaag	this work
R55_T2for	ttctgcagccatatccaatatatctggaatc	this work
R55_T2for2	ttctgcagatagatagagggaaatttgac	this work
R55_T2for3	ttctgcagaaagatgggttagcctagtg	this work
R55_T2for4	ttctgcagacagaactagattccagtattgg	this work
R55_T2rev	aagaattcctctgttatctcggatcgttc	this work
R55_T2rev1	ttgaattcctcaataccctcccaattcc	this work
R55_T2rev2	ttgaattctatctatctggaatttaaagctg	this work
R55_T2rev3	ttgaattccaattcctatccacataac	this work
R55_001Ndefor	ggccatatgagtctaccaacagagcgatg	this work
R55_001XhPErev	aagaattcctgcagctcgagtcacacctcgtcgctatgtgtc	this work
R16_T1rev	ttctgcagacctctcctattccaaaaactc	this work
IP40/R16_T1rev	ttctgcagacttttgaactgctggttgattaattc	this work
pBRPst	gagctgaatgaagccataccaaacgac	[1]
pBRBgl	ttaccatctggccccagtgctgc	[1]
pUCfor21	cagggttttcccagtcacgac	[2]
cat3	agtgataataagcggatgaatgg	[2]
R55_T2delfor	aataagagcatcaagattccagatagatagagggaaatttgacaaattccGTGTAGGCTGGAGCTGCTTC	this work
R55_T2delrev	ttcgagcttcggatctagcagatctagccatcgctctgttggtagactcacATATGAATATCCTCCTTAGTTC	this work
R55_001delstop	tccccagtttcgccaattcagtggccgctacagatgctgtcatgttgtcaGTGTAGGCTGGAGCTGCTTC	this work
R55_001delstart	gagggtattgaggtgagtctaccaacagagcgatggctagatctgctagaCATATGAATATCCTCCTTAGTTC	this work
R55_T1delfor	caagtatgttccagcgagacatcttataccattccatccaaatctgtaaaGTGTAGGCTGGAGCTGCTTC	this work
IP40/R16_T1delrev	caatcggttacgttagaagtggctaaattcaacgcttttaccggccggacCATATGAATATCCTCCTTAGTTC	this work
R55_128delfor	gaatgaagttgtttctcaaagcaaaccctgagtaacaaaaaggagcctaaGTGTAGGCTGGAGCTGCTTC	this work
R55_128delrev	caacgatagtgaaaacatcatgtacaggtgcgagagataatatgtataagCATATGAATATCCTCCTTAGTTC	this work
deltraIR55for	atgctgaaagcccttaacaagttatttggtgggcgaagtggagtgatcgaGTGTAGGCTGGAGCTGCTTC	this work
deltraIR55rev	atgtgtttgctgatgccgatgttttcaccggcaatcatgtcgaaggctttCATATGAATATCCTCCTTAGTTC	this work
orf001promforNc	gaccatggtcaataccctcccaattcctatcc	this work
orf001promrevP	aactgcagcatgaaatcctccaaagattgc	this work
rrnBfor	aactcgaggatcctggcggcagtagcgcggtgg	[3]
rrnBrev	aatctagagtcgacacagataaaacgaaaggcccag	this work

Restriction sites are underlined, uppercase indicate the 3’ part of KO oligos annealing to the template plasmid pKD3.

Supplementary Methods. Construction of plasmids

pJKI708: The 2.08-kb *Hind*III fragment carrying the Sm^R/Sp^R cassette of pHP45Ω [4] was inserted into the unique *Hind*III site of the pACYC177 [5]-derivative plasmid, pJKI88 [2], which inactivated the Km^R gene. The original Ap^R marker of pACYC177 was eliminated in pJKI88 by the introduction of a multicloning site into the *Pst*I-*Bgl*I site of the Ap^R gene.
pJKI854: The 9698-bp *Hpa*I fragment of R16a *traI* KO::Cm^R (coordinates of *Hpa*I sites in R16a are 54768 and 66237 bp) was cloned into the *Hinc*II site of pJKI708.
pJKI962: The 118709-119458 bp *Sac*I fragment of R55 was ligated into the *Sac*I site of pJKI708. The plasmid was isolated from a transconjugant.
pJKI963: The 169567-170810/1-1587 bp *Sac*I fragment of R55 was ligated into the *Sac*I site of pJKI708. The plasmid was isolated from a transconjugant.
pJKI964: The 168441-170810/1-353 bp *Bst*YI fragment of R55 was ligated into the *Bam*HI site of pJKI708. The plasmid was isolated from a transconjugant.
pJKI965: The *Bgl*II-*Bam*HI fragment of pJKI962 was deleted.
pJKI966: The *Bgl*II-*Bam*HI fragment of pJKI962 was ligated into the *Bam*HI site of pJKI708
pJKI967: The *Bgl*II-*Bam*HI fragment of pJKI963 was deleted.
pJKI968: The *Hinc*II fragment of pJKI963 was deleted.
pJKI969: The *Hinc*II fragment of pJKI964 was deleted.
pJKI972: After blunting the *Cla*I-digested end by Klenow polymerase in the absence of dNTPs, the *Eco*RI-*Cla*I fragment of pJKI966 was ligated into the *Eco*RI-*Hinc*II site of pJKI708.
pJKI973: pJKI969 was digested with *Eco*RI-*Nde*I, the protruding ends were blunted with Klenow polymerase in the absence of dNTPs and the plasmid was religated.
pJKI974: pJKI969 was digested with *Pst*I-*Nde*I, the protruding ends were blunted with Klenow polymerase in the absence of dNTPs and the plasmid was religated.
pJKI981: The 119000-119327 bp fragment of R55 was amplified from pJKI972 with primers R55_T1for1 and pBRPst, and the amplicon was digested with *Eco*RI-*Pst*I and ligated into the *Eco*RI-*Pst*I site of pJKI708.
pJKI982: The 119000-119224 bp fragment of R55 was amplified from pJKI972 with primers R55_T1for1 and R55_T1rev, and the amplicon was digested with *Eco*RI-*Pst*I and ligated into the *Eco*RI-*Pst*I site of pJKI708.
pJKI983: The 119065-119327 bp fragment of R55 was amplified from pJKI972 with primers R55_T1for2 and pBRPst, and the amplicon was digested with *Eco*RI-*Pst*I and ligated into the *Eco*RI-*Pst*I site of pJKI708.
pJKI984: The 119065-119224 bp fragment of R55 was amplified from pJKI972 with primers R55_T1for2 and R55_T1rev, and the amplicon was digested with *Eco*RI-*Pst*I and ligated into the *Eco*RI-*Pst*I site of pJKI708.
pJKI985: The 97-353 bp fragment of R55 was amplified from pJKI969 with primers R55_T2for and pBRBgl, and the amplicon was digested with *Eco*RI-*Pst*I and ligated into the *Eco*RI-*Pst*I site of pJKI708.
pJKI986: The 97-323 bp fragment of R55 was amplified from pJKI969 with primers R55_T2for and T2rev1, and the amplicon was digested with *Eco*RI-*Pst*I and ligated into the *Eco*RI-*Pst*I site of pJKI708.
pJKI987: The 97-280 bp fragment of R55 was amplified from pJKI969 with primers R55_T2for and T2rev2, and the amplicon was digested with *Eco*RI-*Pst*I and ligated into the *Eco*RI-*Pst*I site of pJKI708.
pJKI997: The 119065-119190 bp fragment of R55 was amplified from pJKI972 with primers R55_T1for2 and R55_T1rev2, and the amplicon was digested with *Eco*RI-*Pst*I and ligated into the *Eco*RI-*Pst*I site of pJKI708.
pJKI998: The 119107-119227 bp fragment of R55 was amplified from pJKI972 with primers R55_T1for3 and R55_T1rev, and the amplicon was digested with *Eco*RI-*Pst*I and ligated into the *Eco*RI-*Pst*I site of pJKI708.
pJKI999: The 119107-119190 bp fragment of R55 was amplified from pJKI972 with primers R55_T1for3 and R55_T1rev2, and the amplicon was digested with *Eco*RI-*Pst*I and ligated into the *Eco*RI-*Pst*I site of pJKI708.
pJKI1000: The 177-323 bp fragment of R55 was amplified from pJKI969 with primers R55_T2for2 and T2rev1, and the amplicon was digested with *Eco*RI-*Pst*I and ligated into the *Eco*RI-*Pst*I site of pJKI708.
pJKI1001: The 208-323 bp fragment of R55 was amplified from pJKI969 with primers R55_T2for3 and R55_T2rev1, and the amplicon was digested with *Eco*RI-*Pst*I and ligated into the *Eco*RI-*Pst*I site of pJKI708.
pJKI1002: The 97-300 bp fragment of R55 was amplified from pJKI969 with primers R55_T2for and R55_T2rev3, and the amplicon was digested with *Eco*RI-*Pst*I and ligated into the *Eco*RI-*Pst*I site of pJKI708.
pJKI1006: The 208-300 bp fragment of R55 was amplified from pJKI969 with primers R55_T2for3 and R55_T2rev3, and the amplicon was digested with *Eco*RI-*Pst*I and ligated into the *Eco*RI-*Pst*I site of pJKI708.
pJKI1007: The 227-300 bp fragment of R55 was amplified from pJKI969 with primers R55_T2for4 and R55_T2rev3, and the amplicon was digested with *Eco*RI-*Pst*I and ligated into the *Eco*RI-*Pst*I site of pJKI708.
pJKI1008: The 227-323 bp fragment of R55 was amplified from pJKI969 with primers R55_T2for4 and R55_T2rev1, and the amplicon was digested with *Eco*RI-*Pst*I and ligated into the *Eco*RI-*Pst*I site of pJKI708.
pJKI1011: The *mobI* gene of R55 was amplified with primers R55_T2for and R55_001XhPErev, and the amplicon was digested with *Eco*RI-*Nde*I and ligated into the *Eco*RI-*Nde*I site of pJKI969.
pJKI1012: The 97-410 bp fragment of R16a was amplified with primers R55_T2for and R55_T2rev, and the amplicon was digested with *Eco*RI-*Pst*I and ligated into the *Eco*RI-*Pst*I site of pJKI708.

pJKI1021: The *mobI* gene (334-874 bp fragment) of R16a was amplified with primers R55_001Ndefor and R55_001XhPErev and the amplicon was ligated into the *Sma*I site of pBluescript SK to generate pJKI1010. The original start codon (GTG) was changed to ATG by the R55_001Ndefor primer. The rrnB T1 terminator was amplified from pKK223-3 [6] with primers rrnBfor – rrnBrev and cloned into the *Sma*I site of pBluescript SK to construct pJKI941. The rrnB T1 terminator fragment was transferred from pJKI941 into the *Bam*HI-*Xba*I site of pJKI888 [3] leading to pJKI945. Then, the *mobI* gene was excised from pJKI1010 as an *Nde*I-*Bam*HI fragment and ligated into the *Nde*I-*Bam*HI site of pJKI945 (*acaCD* cassette was replaced by *mobI*) leading to pJKI1019. The Sm^R/Sp^R cassette of pHP45Ω [4] was then inserted into the *Hind*III site of the Km^R gene in pJKI1019 to create pJI1021.
pJKI1045: The Mob 2 region of R55 was excised from pJKI972 as a *Sma*I fragment and ligated into the *Sma*I site of the Km^R gene in pJKI1021.
pJKI1051: The 118978-119327 bp Mob 2 region of R55 was excised from pJKI972 as a *Xba*I-*Pst*I fragment and ligated into the *Xba*I-*Pst*I site of pEMBL19 [7] to generate pJKI1047, from which the Mob 2 region was transferred into the *Bam*HI-*Hind*III site of the single copy plasmid pBeloBac11 (NEB).
pJKI1053: The 119169-119224 bp fragment of R55 was amplified from pJKI972 with primers R55_T1for4 and R55_T1rev using Taq polymerase, and the amplicon was digested with *Eco*RI-*Pst*I and ligated into the *Eco*RI-*Pst*I site of pJKI708.
pJKI1054: recHSfor and recHSrev oligos representing the 119191-119224 bp fragment of R55 were annealed and ligated into the *Eco*RI-*Pst*I site of pJKI708.
pJKI1056: The 119169-119224 bp fragment of R55 (RecHS) was amplified from pJKI972 with primers R55_T1for4 and R55_T1rev using Taq polymerase, and the amplicon was digested with *Eco*RI-*Pst*I and ligated into the *Eco*RI-*Pst*I site of pBluescript SK, leading to pJKI1055. RecHS was then transferred from pJKI1055 with *Bam*HI-*Hind*III into the respective site of pBeloBac11.
pMSZ952: The non-coding upstream region of *mobI* was amplified from R55 (170632-170810/1-321 bp) with primers orf001promforNc - orf001promrevP. The amplicon was cut with *Nco*I-*Pst*I and ligated into the corresponding site of pJKI990 [3].

References

1. Kiss, J. *et al.* Transposition and target specificity of the typical IS*30* family element IS*1655* from *Neisseria meningitidis*. *Mol. Microbiol.* **63,** 1731–1747 (2007).
2. Kiss, J. & Olasz, F. Formation and transposition of the covalently closed IS*30* circle: the relation between tandem dimers and monomeric circles. *Mol. Microbiol.* **34,** 37–52 (1999).
3. Kiss, J. *et al.* The master regulator of IncA/C plasmids is recognized by the *Salmonella* Genomic Island SGI1 as a signal for excision and conjugal transfer. *Nucleic Acids Res.* **43,** 8735–8745 (2015).
4. Prentki, P. & Krisch, H. M. *In vitro* insertional mutagenesis with a selectable DNA fragment. *Gene* **29,** 303–313 (1984).
5. Rose, R. E. The nucleotide sequence of pACYC177. *Nucleic Acids Res.* **16,** 356 (1988).
6. Brosius, J. & Holy, A. Regulation of ribosomal RNA promoters with a synthetic lac operator. *Proc. Natl. Acad. Sci. U. S. A.* **81,** 6929–6933 (1984).
7. Dente, L., Cesareni, G. & Cortese, R. pEMBL: A new family of single stranded plasmids. *Nucleic Acids Res.* **11,** 1645–1655 (1983).
